# Neocortical Rebound Depolarization Enhances Visual Perception

**DOI:** 10.1371/journal.pbio.1002231

**Published:** 2015-08-14

**Authors:** Kenta Funayama, Genki Minamisawa, Nobuyoshi Matsumoto, Hiroshi Ban, Allen W. Chan, Norio Matsuki, Timothy H. Murphy, Yuji Ikegaya

**Affiliations:** 1 Laboratory of Chemical Pharmacology, Graduate School of Pharmaceutical Sciences, The University of Tokyo, Bunkyo-ku, Tokyo, Japan; 2 Center for Information and Neural Networks, National Institute of Information and Communications Technology, Suita City, Osaka, Japan; 3 Graduate School of Frontier Biosciences, Osaka University, Suita City, Osaka, Japan; 4 Department of Psychiatry, University of British Columbia, Vancouver, British Columbia, Canada; McGill University, CANADA

## Abstract

Animals are constantly exposed to the time-varying visual world. Because visual perception is modulated by immediately prior visual experience, visual cortical neurons may register recent visual history into a specific form of offline activity and link it to later visual input. To examine how preceding visual inputs interact with upcoming information at the single neuron level, we designed a simple stimulation protocol in which a brief, orientated flashing stimulus was subsequently coupled to visual stimuli with identical or different features. Using in vivo whole-cell patch-clamp recording and functional two-photon calcium imaging from the primary visual cortex (V1) of awake mice, we discovered that a flash of sinusoidal grating per se induces an early, transient activation as well as a long-delayed reactivation in V1 neurons. This late response, which started hundreds of milliseconds after the flash and persisted for approximately 2 s, was also observed in human V1 electroencephalogram. When another drifting grating stimulus arrived during the late response, the V1 neurons exhibited a sublinear, but apparently increased response, especially to the same grating orientation. In behavioral tests of mice and humans, the flashing stimulation enhanced the detection power of the identically orientated visual stimulation only when the second stimulation was presented during the time window of the late response. Therefore, V1 late responses likely provide a neural basis for admixing temporally separated stimuli and extracting identical features in time-varying visual environments.

## Abstract 2

A study of mice and humans shows that prior activity in the visual cortex induces a long-delayed depolarization that enhances perception of subsequent visual stimuli if these are identical to the previous one, thereby extracting invariant visual features from the constantly changing visual world.

## Author Summary

Animals are constantly exposed to a visual world that varies over time. To examine how the visual cortex integrates visual information that is temporally spaced, we monitored neuronal activity of the primary visual cortex (V1) using single- and multicell recording techniques. We discovered that a brief visual stimulus induced an early, transient activation as well as a delayed reactivation of V1 neurons in mice and humans. Notably, this reactivation of visual cortex conveyed information about stimulus orientation: presentation of a second visual stimulus during this reactivation enhanced the V1 response specifically when the orientations of the two stimuli were identical. Behavioral tests in mice and humans revealed that the ability to detect visual stimuli was also enhanced when the second stimulus was presented during the time window of V1 reactivation. Because animals extract visual information from an environment in constant change, the modulation of visual responses through cortical reactivation might be a strategy commonly used in the visual system.

## Introduction

The primary visual cortex (V1) has been used as an experimental model to study cortical responses to sensory input. V1 receives direct synaptic inputs from the lateral geniculate nucleus (LGN) of the thalamus and provides the output of its computation to higher-order cortical areas [1,2]. This route, commonly known as the feed forward pathway, contributes to the hierarchical neural processing of specific visual features, such as orientation, direction, color, and motion. Classical visual processing models consider V1 as a passive relay station for visual information; that is, V1 encodes instantaneous information by transiently responding to the present stimulus feature. However, recent evidence has demonstrated that V1 activity persists over time [3–7] and even propagates throughout the V1 network [8,9]. This complex activity is likely associated with the representation of reward timing [4,5], iconic memory [10,11], and working memory [12–14]. Indeed, reverberatory neuronal activity within neocortical circuitry has been proposed as a potential mechanism for short-term storage of information [15,16].

How does V1 encode the external world while under a constant flow of visual stimuli? The measurement of cortical dynamics has revealed that V1 response tuning evolves with time [17], during which it may interfere with later V1 information [18]. Indeed, preceding visual stimuli are reported to modulate visual perception after brief stimulus-onset asynchrony (SOA) [19–22]. Therefore, poststimulus V1 activity appears to intermingle with the subsequent visual information, which produces a complex output [23–25].

In this study, we discovered a novel V1 activation pattern in nonanesthetized mice; in virtually all V1 neurons, an oriented flashing light–induced biphasic membrane voltage (*V*_*m*_) response that consisted of an early, transient depolarization and a late, slow depolarization. The late response exhibited high orientation selectivity, which indicates that V1 maintains the information of a recent stimulus with high fidelity for some time. Flash-induced late response was also observed using electroencephalogram (EEG) recordings in humans, suggesting that a long-delayed V1 reactivation prevails in mammals. To understand the effect of the late response on the upcoming visual input, we paired a flashing stimulus to another visual stimulus with a time lag. Flashes modulated the V1 response to the subsequent input in an orientation-selective manner. The flash-induced selective modulation was also replicated in the psychophysical parameters of mice and humans.

## Results

### V1 Late Responses

**Fig 1:**
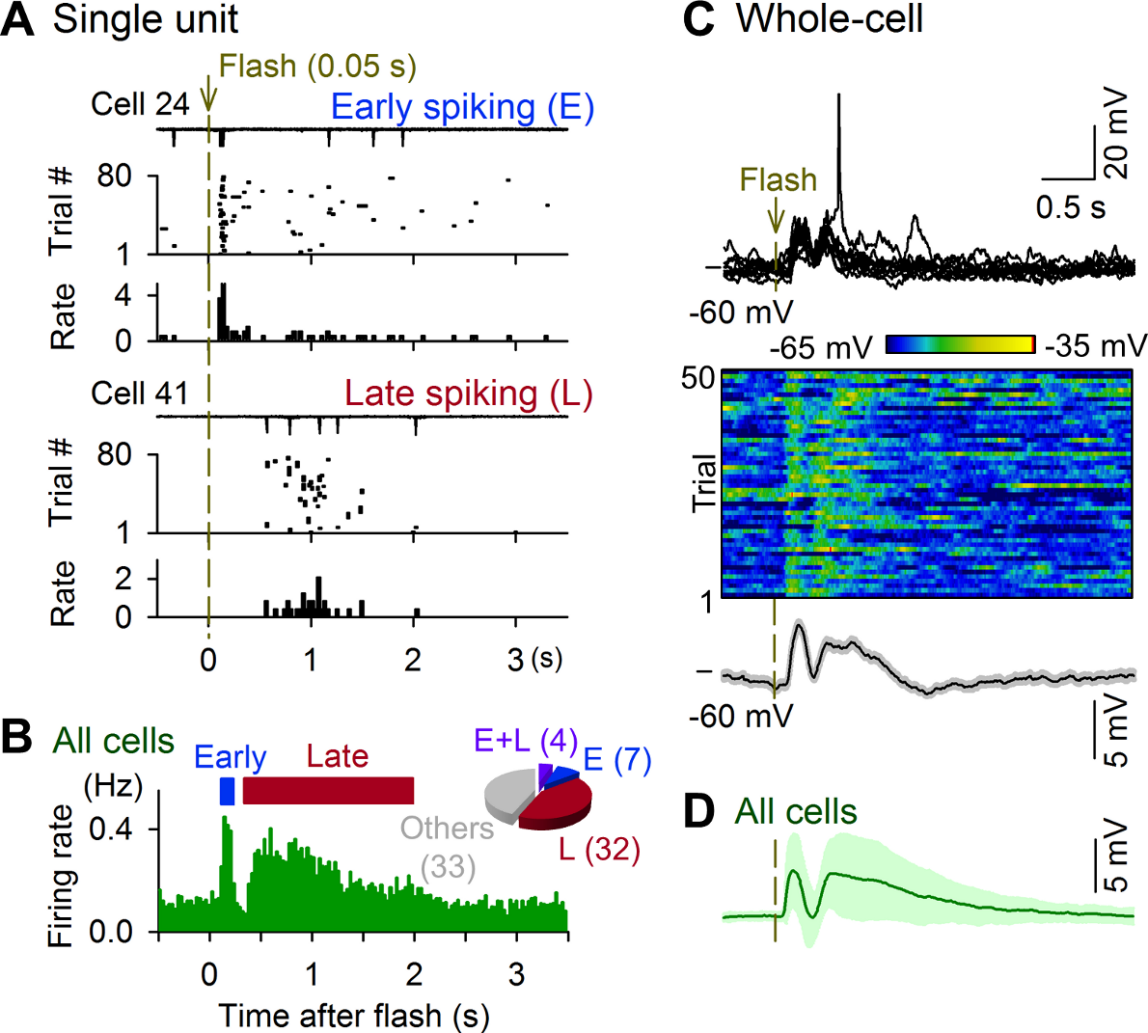
Flash-evoked biphasic responses in mouse V1 neurons. Cell-attached recordings (A and B) and whole-cell recordings (C and D) were acquired from V1 L2/3 neurons in awake, head-restricted mice whose contralateral eyes were presented 0.05-s full-field grating flashes at pseudorandom intervals of 8–10 s for 80–200 trials. (A) Raw traces of cell-attached recordings at 10 consecutive trials, the spike raster plots of the 80 trials and their periflash time histograms of the firing rates for two typical neurons. Cell 24 (S1 Data) fired action potentials with short latencies, whereas cell 41 (S1 Data) fired action potentials with longer latencies. (B) Data from 76 cells from 58 mice are pooled. The inset pie chart indicates the distribution of cells with early spiking (E), late spiking (L), and no activity change (others). (C) The top raw traces show *V*_*m*_ responses in 10 consecutive trials in a representative neuron. *V*_*m*_ responses for 50 trials in the same neuron (middle pseudocolored map) were averaged in the bottom trace. The gray area indicates the standard errors of the mean (SEMs). (D) Mean ± SD of the subthreshold *V*_*m*_ responses of 34 cells from 30 mice.

We monitored the spiking activity of V1 layer (L) 2/3 neurons of P35–P44 mice using the cell-attached recording technique (Fig 1A) and applied a brief flashing stimulus (17–50 ms) of a full-field grayscale sinusoidal grating with one of four orientations (0°, 45°, 90°, and 135°) to the eye contralateral to the recording site. As previous reports have demonstrated that L2/3 neurons fire sparsely [26–30], 56.5% of V1 neurons (43 of 76 cells) exhibited a significant increase in their firing rates in response to the grating flashes (defined by a criterion of *p* < 0.05 versus the baseline firing rates, *Z* test for comparison of two counts [31]). The responses were classified into two types; the first type of responses was spikes immediately (< 0.3 s) after the stimulus onset (early spiking, Fig 1A top), whereas the second type was spikes with latencies longer than 0.4 s (late spiking, Fig 1A bottom). In the pooled data, the population firing rates exhibited two distinct peaks that corresponded to the first and second types of spikes; for individual responsive neurons, the mean firing rates during the early and late responses were 1.27 ± 0.91 Hz and 0.28 ± 0.19 Hz, respectively (mean ± standard deviation [SD] of 11 and 36 neurons). Late-spiking neurons were numerically dominant (Fig 1B, inset). Thus, we defined the early and late responses as activity that occurred between 0–0.3 s and 0.4–2 s, respectively.

To investigate the subthreshold *V*_*m*_ dynamics that underlie the biphasic spike responses, we conducted whole-cell current-clamp (*I* = 0) recordings from V1 neurons (S1A and S1B Fig). In the typical neuron shown in Fig 1C, a grating flash reliably induced early and late depolarization responses. Remarkably, we observed similar biphasic *V*_*m*_ responses in all 28 recorded neurons (S1C Fig), irrespective of their firing types, including nonspiking neurons (S1D Fig). The early depolarization was transient and peaked at latencies of < 0.3 s, whereas the late depolarization was more persistent and peaked at approximately 0.4−2.0 s. On average, the peak amplitudes of the early and late depolarizations were 6.7 ± 4.2 and 6.4 ± 4.4 mV (mean ± SD of 28 cells), respectively, and were correlated with each other (S1C Fig left). The area under curves of individual *V*_m_ traces during a late period of 0.4–2.0 s (late area) was correlated with their peak amplitudes (S1C Fig middle). Therefore, we quantified both early and late responses using their peak amplitudes in the following analyses. The areas of late responses were not correlated with their peak latencies (S1C Fig right). Thus, the latencies did not affect the magnitude of late responses. This fact also validates our choice of the time window for late *V*_m_ responses (0.4–2.0 s).

**Fig 2:**
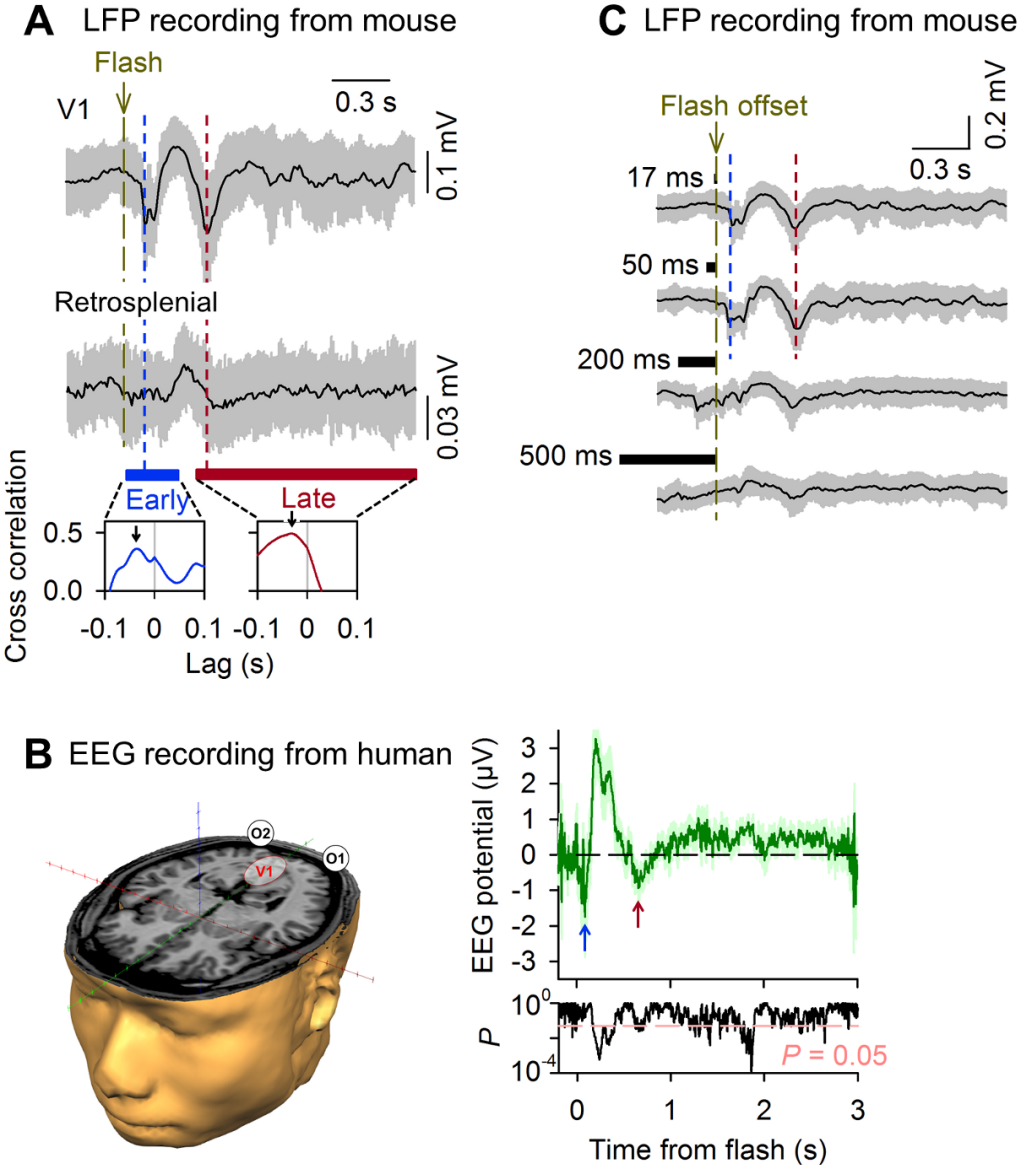
Biphasic responses of field potentials in mouse and human visual cortex to grating flashes. (A) LFPs were recorded from L2/3 of the V1 and retrosplenial cortex, while a full-field grating flash was presented to the contralateral eye of an awake mouse. Two negative potentials appeared after a flash. The gray areas indicate the SDs. The arrows in the bottom cross correlograms indicate the peak offsets, which show that early and late responses occurred earlier in V1 than in the retrosplenial cortex. (B) Human EEGs were recorded from O1 and O2, indicated in the left schematic. ERPs in responses to grating flashes are shown as mean ± SD of 10 participants. The arrows indicate early and late negative potentials. The bottom plot represents the *p*-values from the prestimulus baseline at the corresponding time points, indicating the presence of early and late responses. (C) Flashes with shorter durations induced more evident late responses in mouse V1 LFPs.

The fact that late depolarizations occurred in all recorded neurons suggests that late visual responses represent a global phenomenon that involves the entire V1 cortex. To confirm this possibility, we recorded local field potentials (LFPs), which reflect the compound activity of multiple neurons surrounding the tip of a recording electrode [32]. We found that LFPs in V1 L2/3 responded reliably to a grating flash with biphasic negative fluctuations (Fig 2A). The response signal, if any, was less evident in LFPs recorded from the retrosplenial cortex, a more anterior brain region. We also recorded voltage dynamics of the neocortical surface. We loaded the cerebral surface with RH-1692, a voltage-sensitive dye (VSD), and monitored the spatiotemporal patterns of flash-evoked activity [33]. As expected by the LFP data, early cortical VSD responses were observed in V1 (S2 Fig). Then, the VSD signal decreased transiently, producing a transitional period. After approximately 0.4 s, the late VSD responses also arose at V1. Therefore, similar to *V*_m_ responses in patch-clamp recordings, the VSD signal in V1 was biphasic. We extended the field potential work to visual responses in humans. We recorded EEG from 10 adult participants and measured visual event-related potentials (ERPs) at O1 and O2, according to the international 10/20 coordinate convention [34]. Human ERPs in response to grating flashes were also biphasic; an early and late negative reflection peaked around 0.15 s and 0.7 s, respectively, after a grating flash (Fig 2B).

Previous studies have also reported a specific form of late, slow activation of the rat V1 [4,5] and the mouse primary somatosensory cortex [35]; however, these responses emerged as a result of sensory reinforcement learning and were not observed in naïve animals. There is also a study that has reported biphasic responses in naïve cat visual cortex [36]; however, the latency and the duration of this late response was much shorter. By contrast, our flash-evoked late V1 responses occurred in naïve animals and had a much longer latency and duration. Therefore, they represent novel V1 dynamics. This discrepancy most likely occurs as a result of the difference in the features of visual stimuli. Indeed, the durations of flashes were critical [7]; we failed to observe evident long-delayed LFP activity at flash durations of more than 200 ms (Fig 2C). Moreover, we used full-field flashes, which might recruit synaptic inputs from both classical and nonclassical visual receptive fields. It should also be noted that flash-induced late response has a much longer duration than the well-known OFF response that has been described in other studies [37].

### Orientation Selectivity of Late V1 Response

**Fig 3:**
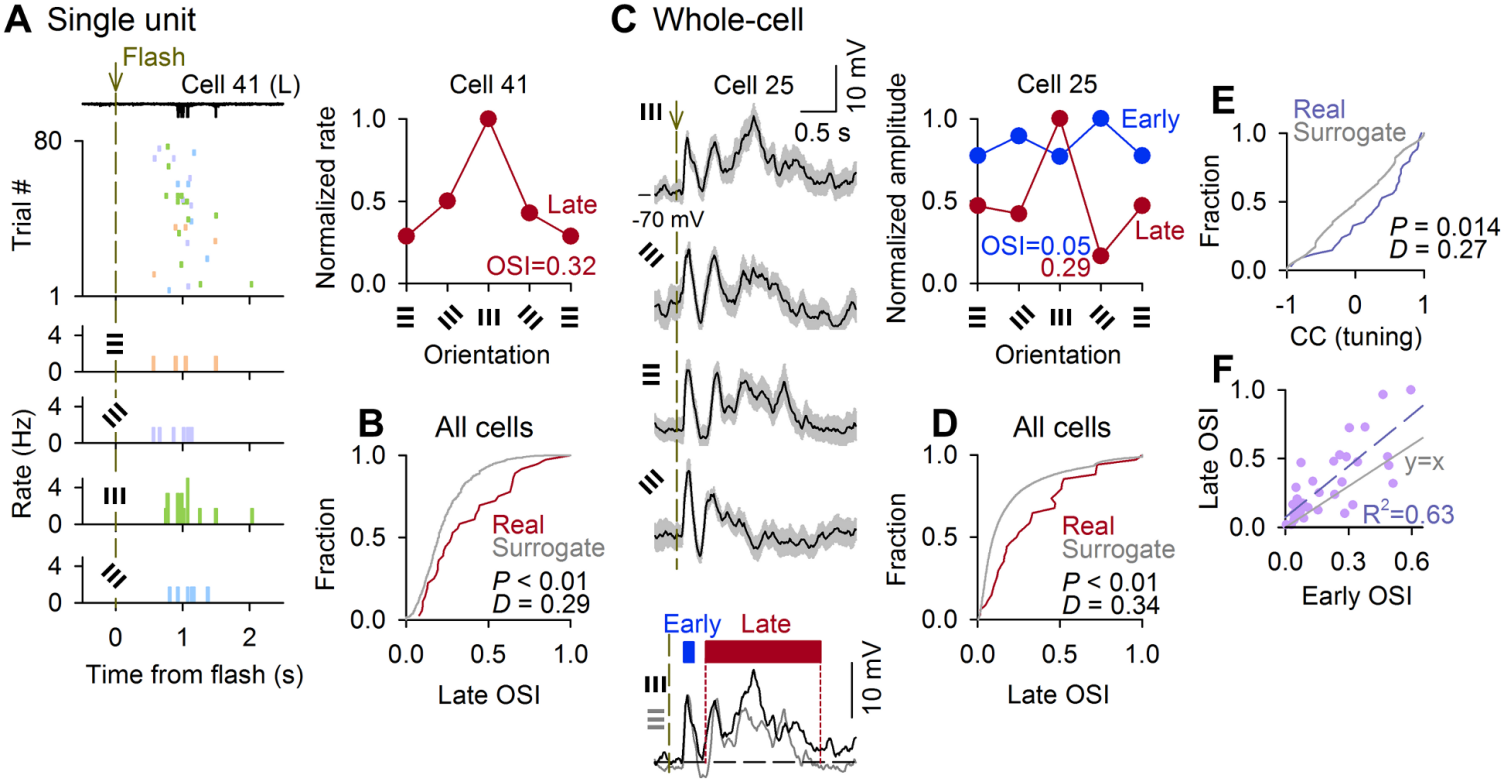
Orientation selectivity of late V1 response. (A) Left, raw traces in 10 trials of cell-attached recordings, raster plots of spike responses in 80 trials, and periflash time histograms of the firing rates for four orientations of the grating flash stimulation in a representative neuron (S3 Data). The orientations are shown in different colors. Right, the orientation tuning curve of the same neuron. The evoked spike counts were normalized to the maximum. (B) The cumulative probability distribution of the OSIs of the 36 late-spiking cells (Real) was compared with its chance distribution (Surrogate) that was obtained by 1,000 random shufflings of the stimulus trials. The real OSIs were biased rightward compared with the surrogate OSIs (*p* = 3.3 × 10^−3^, *D* = 0.29, Kolmogorov-Smirnov test). (C) Left traces represent the mean ± SD of subthreshold *V*_*m*_ responses of an example cell (S3 Data) to grating flashes with four orientations. The right plot indicates the orientation tuning curves of the mean amplitude of the early and late *V*_*m*_ depolarizations of the same neuron. (D) The cumulative fraction of the OSIs in the late *V*_*m*_ responses were biased rightward compared with their surrogate OSIs (*n* = 34 cells, *p* = 2.7 × 10^−9^, *D* = 0.66, Kolmogorov-Smirnov test). (E) The correlation coefficients between the early and late tuning curves for individual cells were higher compared with their chance values calculated by random trial-shuffling of the early responses (*n* = 34 cells, *p* = 0.014, *D* = 0.27, Kolmogorov-Smirnov test). (F) Scatter plots of the OSIs in early and late responses for individual cells. Each dot indicates a single cell. The gray line is the diagonal, and the dash line is the best linear fit.

The amplitudes of both early and late responses increased at higher contrasts of flash gratings (S3 Fig). Thus, it is feasible that the late responses encode the orientation of flashing stimuli [36]. We measured the orientation selectivity, which is a characteristic of V1 neuron responses [38–41]. Grating flashes with various orientations induced different changes in the late spike rates (Fig 3A and S4A Fig). We calculated the orientation selectivity index (OSI) for each late-spiking neuron. On average, the OSIs were 0.37 ± 0.25 (mean ± SD of 36 cells). To evaluate the statistical significance of OSIs, we compared them with the chance distribution obtained from the trial-shuffled surrogate data (Fig 3B). Overall, the OSIs exhibited significantly higher values than chance, which indicates that the late-spiking responses were orientation-selective (*p* = 3.3 × 10^−3^. *D* = 0.29, *n* = 36 cells, Kolmogorov-Smirnov test). Late subthreshold *V*_*m*_ responses were also significantly orientation-selective (Fig 3C and 3D, *p* = 2.7 × 10^−9^, *D* = 0.66, *n* = 34 cells, Kolmogorov-Smirnov test). Their OSIs were lower compared with the late spike responses (S4B and S4C Fig, *p* = 5.0 × 10^−3^, *t*_19_ = 3.17, *n* = 20 cells, paired *t* test), consistent with many previous reports about orientation selectivity of *V*_m_ responses [42–44].

Because the early responses were also orientation-selective, we focused on the tuning properties of the early and late responses. We computed the correlation coefficients between the early and late *V*_*m*_ tuning curves of each cell and compared the pooled data to the chance-level distribution of the correlation coefficients in their trial-shuffled surrogates. The correlation coefficients were significantly higher compared with chance, which indicates that the early and late *V*_*m*_ responses of each neuron had a similar orientation tuning (Fig 3E, *p* = 0.014, *D* = 0.27, *n* = 34 cells, Kolmogorov-Smirnov test). Moreover, the OSIs of late responses were positively correlated with the OSIs of early responses (Fig 3F, *R* ^2^ = 0.61, *p* = 1.2 × 10^−4^, *t*_17_ = 4.94, *t* test for a correlation coefficient). Note that neither early nor late OSIs depended on firing rates (S4A Fig, *p* = 0.490, *R* ^2^ = 0.01). We thus conclude that late responses conveyed selective information of visual stimuli.

We further confirmed flash-induced responses using two-photon calcium imaging. We loaded V1 L2/3 neurons with Fura 2 by pressure-applying its acetoxymethyl ester (AM) derivative (S5A Fig). The amplitude of a spike-elicited calcium elevation (|Δ*F*/*F*|) was nearly linear with the number of action potentials involved in the calcium event (S5B Fig). Note that our imaging system was able to resolve two action potentials at an interspike interval of less than 400 ms (S5C Fig), allowing us to classify early and late spiking neurons. We imaged spike-triggered calcium events en masse from 64.6 ± 6.04 neurons per video (mean ± SD of nine videos from nine mice) with a single-cell resolution at five frames per s (S5D Fig). In the example neuron shown in S5E Fig, the amplitudes of the Δ*F*/*F* responses evoked by grating flashes exhibited orientation selectivity. Of the 581 neurons, 323 (56%) neurons were responsive to flashes, and the preferred orientations were uniformly distributed (S5F Fig). Because early spiking responses occurred around 0.1–0.2 s after a flash, they would be reflected in a rapid Δ*F*/*F* increase in the first video frame (0.2 s) after the stimulus. According to this definition, we estimated that early spiking neurons contributed 10.0% (58 out of a total of 581 cells), consistent with patch-clamp recording data showing that the majority of flash-responsive neurons are of the late-spiking type (Fig 1B inset and S1D Fig). Therefore, we assumed that most Δ*F*/*F* responses reflected putatively late spikes. Although they may overlap with the early-spiking component, the orientation tuning properties were approximately congruent between the early and late responses (see Fig 3E), and thus, the Δ*F*/*F* response tuning is still thought to reflect the late-spiking tunings. Consistent with this notion, the distribution of OSIs in the Δ*F*/*F* responses was similar to the late-spiking responses obtained by patch-clamp recordings (S5G Fig, *p* = 0.497, *D* = 0.15, Kolmogorov-Smirnov test) and was higher than that of their surrogate data (*p* = 2.3×10^−6^, *D* = 0.15, *n* = 323 cells).

### Flash-Modulated V1 Response

Because the late response has a long latency, it may interact with a subsequent visual stimulus. We tested this idea by recording the Δ*F*/*F* responses to grating stimuli that moved for 2 s toward one of eight directions (0°, 45°, 90°, 135°, 180°, 225°, 270°, and 315°), which were presented alone (Drift-only trials) or 0.5 s after grating flashes (Flash+Drift trials). To minimize photobleaching and phototoxicity, we did not test all possible combinations of the flash orientations and the drifting grating directions; instead, we fixed the grating flash orientation to 0° (vertical orientation; vFlash) and reduced the total imaging period (S6A Fig). We compared the Δ*F*/*F* responses between Flash+Drift and Drift-only trials and examined how the preceding vFlash (prime) modulated the Δ*F*/*F* responses to subsequent drifting gratings (target). The combinational pattern of a vFlash stimulus and a drifting grating was described as a Δorientation, which represents the orientation difference between vFlash and the drifting gratings and comprised a value of −45°, 0°, 45°, or 90° (= −90°). In Drift-only trials, Δorientation indicates the difference between 0° and the orientations of drifting gratings (i.e., the absolute orientation).

S6B Fig summarizes the data from a representative neuron. For each Δorientation in Drift-only and Flash+Drift trials, we statistically judged whether the neuron responded, i.e., whether the Δ*F*/*F* amplitude was significantly higher compared with the baseline Δ*F*/*F* fluctuation (*p* < 0.05, *n* = 10−18 trials, paired *t* test). The significant responses are marked by dark red boxes below the tuning plot. Three other examples are shown in S6C Fig. We pooled the data from the 581 neurons (S6D Fig). For each Δorientation, we compared the number of cells that exhibited significant Δ*F*/*F* in Drift-only trials to the number of significant cells in Flash+Drift trials. Notably, the number of significantly responsive cells increased at Δorientation = 0°, where the orientations of vFlash and drifting gratings were matched. The number of responsive cells did not increase at the other Δorientations. Thus, two sequential stimuli with the same orientation activated V1 neurons more efficiently compared with stimuli with different orientations. By focusing on individual cells that were activated under the iso-orientation condition, we analyzed their intrinsic orientation preferences. Flash-induced response enhancement was more evident in cells whose preferred orientations were different from the stimulus orientation (S6E Fig). These data indicate that a flash recruited otherwise irresponsive cells (due to their cross orientation preferences) to a subsequent stimulus with the same orientation as the flash.

**Fig 4:**
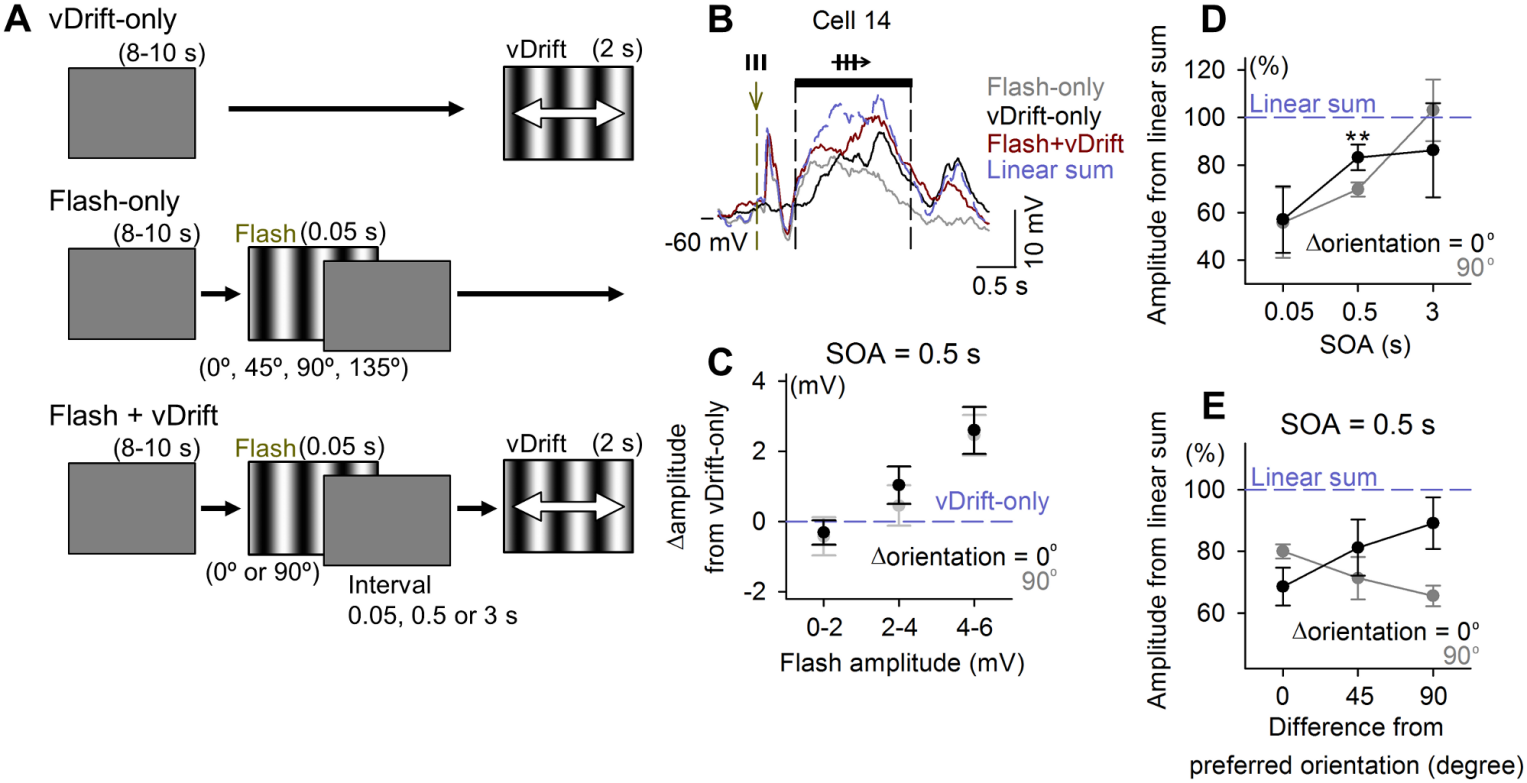
Flash-induced facilitation of *V*_*m*_ response to subsequent visual information. (A) A schematic showing the visual stimulation protocol without (vDrift-only, control) and with (Flash+vDrift) 0.05-s full-field grating flashes followed by 2-s drifting vertical gratings (vDrift) with various SOAs. vDrift-only and Flash+vDrift trials were compared to measure how the preceding flash modulated the *V*_m_ response to vDrift. In some of the trials, Flash was presented alone (Flash-only) to record flash-induced responses. (B) Mean subthreshold *V*_m_ responses of a representative whole-cell recorded neuron (S4 Data). The timing and pattern of visual stimulation are indicated above the traces. The linear sum of responses was calculated by a simple addition of Flash-only and vDrift-only responses. (C) Means ± SEMs of the amplitudes of the Flash+vDrift responses relative to vDrift-only responses at a SOA of 0.5 s were plotted against the amplitudes of the Flash-only responses. The stimulus combination was described as Δorientation, which indicates the orientation difference between the Drift and vFlash. Black and gray symbols indicate Δorientation = 0° and 90°, respectively. (D) Means ± SEM of the amplitude of the Flash+vDrift response relative to the linear sum at SOA of 0.05, 0.5, and 3 s. Black and gray symbols indicate Δorientation = 0° and 90°, respectively (0.5 s: **p = 5.0 × 10^−3^ versus Δorientation = 90°, *t*_25_ = 3.07, *n* = 26 cells from 25 mice, paired *t* test). (E) The data at an SOA of 0.5 s in (D) were divided along the orientation preferences of the neurons.

Previous studies have reported that paired visual stimuli lead to a functional adaptation of neuronal responses to the target [45,46]. In other words, visual cortical neurons decrease their responsiveness to repeated stimuli. Calcium imaging did not allow us to strictly quantify the response amplitude, and we could not determine whether the observed changes are adaptation (desensitization) or priming (sensitization). To quantify the effect of flashes in more details, we returned to patch-clamp recordings of subthreshold *V*_m_ responses. In these experiments, the drifting grating orientation was fixed to vertical (0°, 180°; vDrift), and the orientations of the preceding flashes varied across four orientations (0°, 45°, 90°, or 135°) in a pseudorandom order (Fig 4A). First, the SOA was set to be 0.5 s (Fig 4B). We compared the amplitudes of *V*_m_ responses to a combination of flash and vDrift stimuli (Flash+vDrift) with those of the responses to vDrift alone (vDrift-only). On average, the absolute amplitudes of Flash+vDrift responses were larger than those to vDrift-only responses (*p* = 0.012, *t*_51_ = 2.60, paired *t* test); however, for individual neurons, the amplitude relations depended on the amplitudes to responses to Flash alone (Flash-only, Fig 4C). That is, when a neuron exhibited a large depolarization in Flash-only trials (>2 mV), then the depolarization in Flash+vDrift trials was more increased compared to vDrift-only responses. On the other hand, when a neuron exhibited a small depolarization in Flash-only trials (<2 mV), the Flash+vDrift response amplitude was nearly comparable to the vDrift-only response amplitude. To further examine this effect, we employed a new analysis in which we compared Flash+vDrift responses with the linear summation of the Flash-only response and the vDrift-only response (Fig 4D). We found that this augmentation occurred below the value of simple arithmetic summation of two responses. That is, individual responses to Flash-only and vDrift-only stimuli were sublinearly integrated in Flash+vDrift trials (Fig 4D). In our experimental conditions, therefore, a flash facilitated the vDrift responses through a sublinear integration of *V*_m_ depolarizations. Notably, their sublinearity differed depending on the orientations of flash gratings and was smaller at Δorientation = 0° than at 90° (Fig 4D). In other words, when two orientations of flash gratings and drifting gratings were matched, the combined responses were less sublinear, thereby exhibiting apparently larger response amplitudes, which is consistent with the flash-induced enhancement in the calcium imaging experiments. This Δorientation-dependent difference was not found at SOAs of 0.05 or 3 s (Fig 4D), suggesting the involvement of the orientation selectivity of flash-induced late responses. We replotted these sublinear behaviors (SOA = 0.5 s) as a function of the difference between their intrinsic orientation preferences and the orientation of the grating stimuli. Flash-induced response sublinearity was the largest in cells whose preferred orientations were identical to the stimulus orientation (Fig 4E). This was also consistent with the results in calcium imaging.

### Flash-Modulated Visual Perception

**Fig 5:**
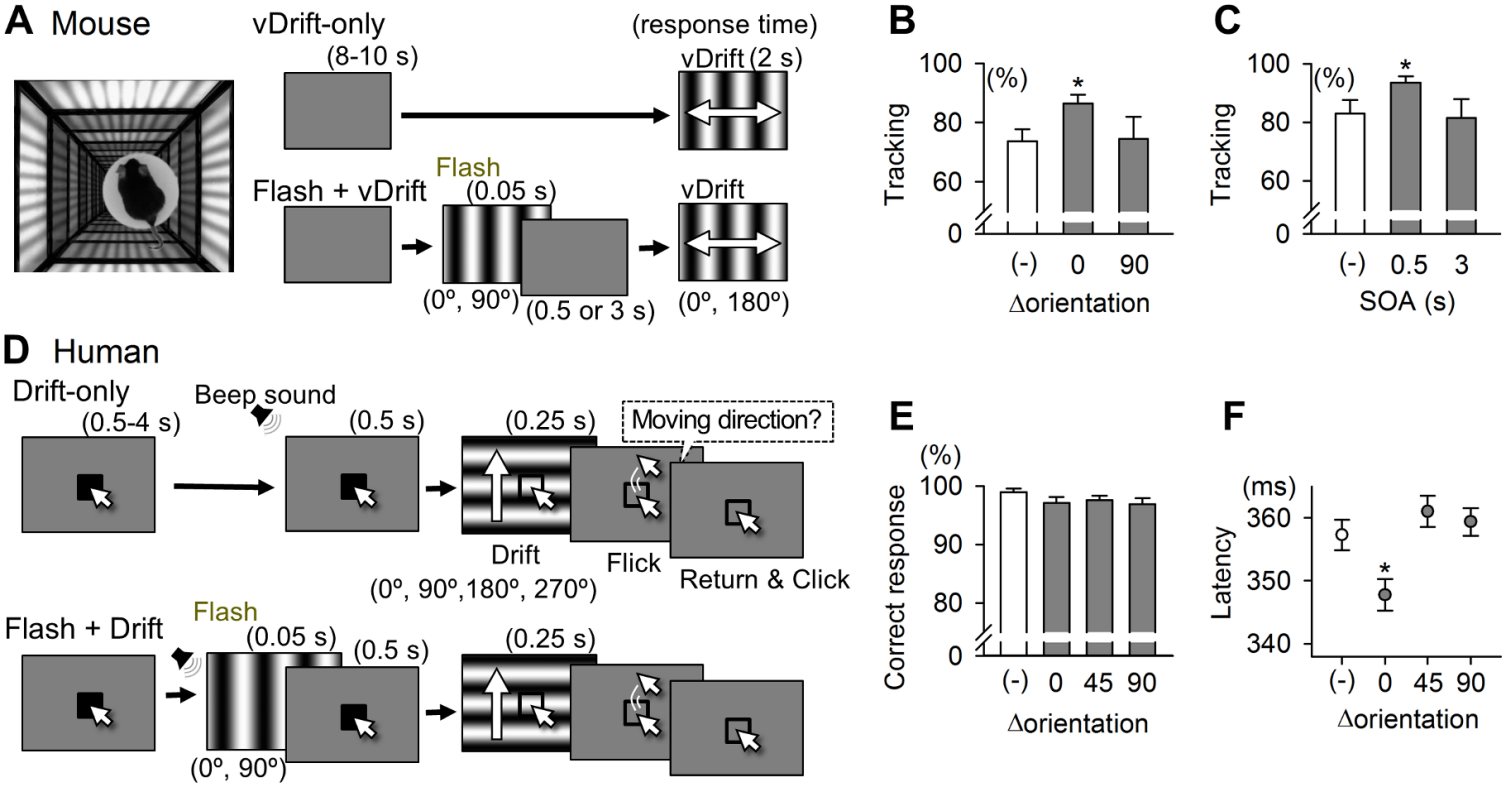
Flash-enhanced visual perception in mice and humans. The visual perception of mice (A–C) and humans (D–F) was examined using visual detection tasks. (A) The left photograph shows the virtual optomotor system in which a mouse was placed on the platform surrounded by four computer screens. When vertical gratings that drifted rightward or leftward (vDrift) were presented for 2 s on the computer displays, the mouse may have reflexively moved its head toward the direction of the motion. The ratio of trials that exhibited this tracking response to all trials was used as a measure of visual detection ability. The right schematics show the visual stimulation protocol without (vDrift-only, control(-)) and with (Flash+vDrift) 0.05-s full-field grating flashes presented 0.5 or 3 s before vDrift. Vertical and horizontal grating flashes were used as Δorientation = 0° and 90°, respectively. (B) Tracking rates of vDrift-only and Flash+vDrift trials at a delay time of 0.5 s. The tracking rates significantly increased at Δorientation = 0° (*p = 0.037, *t*_9_ = 2.45, *n* = 10 mice, paired *t* test). (C) The flash increased the tracking rates only when vDrift was presented with a delay time of 0.5 s, which was comparable to the timing of the Flash-induced late responses (*p = 0.020, *t*_10_ = 2.76, *n* = 11 mice, paired *t* test). (D) Schematic of the behavioral procedure of a visual motion detection task in humans. In each trial, a Drift was presented in one of four motion directions to which the subject was required to respond by flicking a computer mouse. In Flash+Drift trials, flashes were presented with beep sound cues, whereas in Drift-only trials, the sound cues were applied at the same time without flash stimuli. (E) The percentage of correct responses to all relevant trials was comparable between the stimulus conditions. (F) The response latency from the Drift stimulus onset was significantly shorter for the Δorientation = 0° (*p = 0.007, *t*_993_ = 2.71, *n* = 497–498 trials from 11 humans, Student’s *t* test). Error bars represent the SEMs.

Flash-induced modulation of V1 neuronal activity prompted us to evaluate its behavioral consequences. We first measured the visual performance of mice using a virtual optomotor test, which can assess the visual detection ability of naïve mice without behavioral training [47]. A freely moving mouse was placed on the circular platform surrounded by four computer screens on which vertically orientated gratings moved leftward or rightward for 2 s (Fig 5A; vDrift). As a visuomotor reflex, the mouse turned its head in the same direction as the vDrift movement, a behavior that is called a tracking response. The ratio of trials with the tracking responses to the total trials was calculated as the tracking rate and was used as a quantitative measure of visual function. Under the baseline conditions (i.e., vDrift-only trials), the mean tracking rate was 74 ± 13% (mean ± SD of 10 mice). This ratio increased to 86 ± 10% when vertical flashes were presented 0.5 s before vDrift (Fig 5B, Δorientation = 0°; *p* = 0.037, *t*_9_ = 2.45, paired *t* test). This increment was not observed when horizontal flashes (Δorientation = 90°) were coupled (Fig 5B; *p* = 0.92, *t*_9_ = 0.10) or when vertical flashes were presented at an SOA of 3 s (Fig 5C; *p* = 0.69, *t*_10_ = 0.41). In mice that received local injection of 10 μM tetrodotoxin into the V1, flash-induced responses in V1 LFP disappeared (S7A Fig). In these mice, the tracking rate for the vDrift-only trials was reduced to 18 ± 16% (*n* = 4 mice, *p* = 0.026 versus naïve mice, *t*_3_ = 4.13, Student’s *t* test) and was not increased by vertical flashes (S7B Fig). Thus, flash-induced increases in the tracking rates likely depend on V1 late responses.

Finally, we conducted a psychophysical test in humans. The participants were asked to report the motion directions of 0.25-s drifting gratings (0°, 90°, 180°, or 270°) by flicking a computer mouse toward the same direction within 0.70 s (Fig 5D). In Flash+Drift trials, grating flashings at orientations of 0°, 45°, 90°, or 135° were presented 0.5 s before the drifting gratings. The correct response ratio was approximately 100% and was not modulated by grating flashes with either Δorientation (Fig 5E; *p* > 0.05, *n* = 11 humans, *n* = 486−500 trials each, Student’s *t* test). However, the latency of the flicking response was significantly shortened at Δorientation = 0 (Fig 5F; Drift-only: 357.3 ± 54.6 ms versus 0°: 347.8 ± 56.0 ms, mean ± SD; *P* = 0.007, *t*_993_ = 2.71). We did not think that this effect was due to illusory motion perception, because the grating phase of a flash stimulus and the first frame of the following drifting stimulus were identical. However, to examine the possible involvement of motion illusion, we presented two successive flashes at an SOA of 0.5 s with various combinations of the grating phases and asked participants to answer the "felt" motion direction (S8 Fig). Each stimulus condition was repeated for 80 times. As a result, the participants were not able to distinguish the motion direction; the responses were approximately 50% (= the chance level). Thus, two consecutive grating stimuli at an SOA of 0.5 s per se did not induce a motion perception.

## Discussion

We discovered that a brief flashing light evokes long-delayed, slow activation of the mouse V1 network. The late response was observed using different techniques, including patch-clamp recording, LFP recording, VSD recording, and EEG recording, which exclude the possibility of our recording artifact. Importantly, the late response actively interacted with subsequent visual input. This novel phenomenon was heretofore overlooked, probably because past studies tended to record visual responses for shorter terms (up to a few hundreds of milliseconds) than our work, and because we used a short flash of full-field gratings, a stimulus pattern that is not very common in vision research. Another reason for the overlook of the late responses may be a consensus that visual responses occur within a few hundred milliseconds after the onset of the visual stimulus, which might have prevented an attempt to record visual responses for seconds.

There are mainly three candidates for the initiation site of the late response. First, the late activation of V1 circuit might be generated through reverberation of the recurrent circuit within the V1. Theoretically, cortical activity is sustained by local reverberation within a recurrent network [15,16]. Anatomically, L2/3 is enriched with horizontal synaptic connections [48,49] and provides the structural basis of a recurrent circuit. Although V1 L2/3 neurons receive synaptic inputs with various orientation preferences [50], the synaptic connection probability is biased toward a similar orientation preference [51,52]. Recent studies have demonstrated that neurons derived from the same precursor cells are more likely connected and share the same orientation preference [53–55]. These observations suggest the existence of fine-scale subnetworks dedicated to process specific information [56]. We determined that the tuning properties were significantly correlated between the early and late responses. Hence, the neuron population activated by a grating flash is preferentially reactivated at the late phase. The visual cortex may filter visual input information through its specifically wired, reverberatory network [57] and may offer a high orientation tuning during the late response. The second possibility is that the V1 rebound activity arose from subcortical regions, including the lateral geniculate thalamus and the superior colliculus (and even the retina). The lateral geniculate thalamus is anatomically eligible for generating rebound activation, because it contains a recurrent network and receives feedback projections from V1 [37,58]. This anatomy might have led to the reliable observation of late response even in the LFP recording. Finally, top-down inputs from higher-order cortices may also have the ability to induce late responses, as recently reported in the hindlimb somatosensory cortex [59]. However, the latency of the late response in the visual cortex was much longer than that observed in the study, suggesting a more complex mechanism than a simple top-down feedback process.

We speculate that reverberatory activity in V1-recurrent circuits admixes with late-coming feed forward V1 activity. Recent studies have demonstrated that costimulation of the thalamocortical and cortical pathways efficiently depolarizes cortical neurons through nonlinear summation [60,61]. Although a single L2/3 neuron receives variously tuned synaptic inputs irrespective of the orientation preference in the cell’s spike output [50], synaptic inputs over dendritic trees are nonrandomly distributed and are often spatially clustered [62–64]. Thus, synaptic inputs from flashing and drifting gratings may be locally converged and may lead to nonlinear dendritic boost [61,65] when two orientations are matched.

At the network level, a grating flash enhanced (or sublinearly integrated) the V1 responses to subsequent drifting gratings in an orientation-selective manner. In these experiments, we used an SOA of 0.5 so that drifting gratings arrived during the period of flash-evoked late responses. Calcium Δ*F*/*F* responses to the drifting gratings were enhanced only when their orientations were identical to the preceding flashes. The flash-induced facilitation can be explained by two possibilities. First, the priming effect may facilitate the responses to sequential stimuli [66,67]. However, flash-induced response enhancement is not a normal form of priming because it was not a simple mixture of membrane-potential depolarizations. Flash-induced late response and the response to drifting grating were integrated in a sublinear fashion, but more linearly at Δorientation = 0°, suggestive of the partial existence of priming. It also differed depending on preferred orientations of the neurons. The second possibility is that the facilitation occurred through top-down neural processing [68], especially feature-based attention [69,70]. It is well known that attention modulates the responsiveness of neurons that have receptive fields within the attentional loci [71–73], enhancing task performance on late-coming target stimuli [70,74]. Moreover, it is important to note that feature attention in humans is effective at an SOA of approximately 0.5 s [69], consistent with our findings. Developing a psychophysical method to measure the attentional effect in mice may help verify the second possibility. Focusing on individual neurons and their orientation preferences, a flash recruited neurons with shifted-orientation preferences at the Δorientation = 0° condition. In other words, neurons with cross orientated preferences to the flash orientation were less subject to the sublinearity when the responses were integrated. Consistent with this notion, at Δorientation = 90°, neurons with cross orientated preferences to a flash (i.e., iso-orientated with regard to the orientation of the drifting stimulus) exhibited the minimal sublinear property. Thus, flash-induced late responses might function to recruit neurons that are otherwise irresponsive, leading to stronger activation of the V1.

We found that ongoing visual processing and perception were both affected by the immediately preceding visual information in a feature-specific manner; however, we could not directly show the causal contribution of flash-induced delayed depolarizations per se to subsequent visual perception. Optogenetic prevention of the delayed responses [35] is not applicable to our cases; that is, even if optogenetic manipulation is performed only during the delayed activity period, it inevitably affects both flash-induced delayed responses and drifting grating-evoked activity and cannot isolate the effect of the flash responses on visual perception. Therefore, we need to seek a way to specifically diminish the delayed activity without affecting drifting grating-evoked activity.

In this study, we regarded the featured flashes as a model of the initial visual scenes and aimed to separate the effect of suddenly coming and subsequently continuing visual scenes. Hence, we think that, under natural conditions, the pattern-selective late responses observed here may work to facilitate the responses to the passing object, possibly linking our findings to studies on trans-saccadic integration [75–77]. However, two major concerns remain unresolved. First, the late response occurred to flashes with durations of less than 50 ms, whereas natural saccades usually last about 300 ms. Thus, we cannot rule out the possibility that the late response we found is involved in other visual processes than trans-saccadic integrations. Second, although we obtained the behavioral correlates of flash-induced effects on visual function, flashes recruited neurons that were otherwise irresponsive because of the nonpreferred orientation. Therefore, flashes may increase the overall activity level of V1 and diminish the selective responsiveness of individual neurons. According to this notion, the facilitation of V1 activity would decrease the discrimination acuity of the animal, but at the same time, it could increase the sensitivity per se by lowering the visual detection threshold. This possibility must be clarified using a new behavioral paradigm that can distinguish visual detection from visual discrimination.

## Materials and Methods

### Ethical Approval

Animal experiments were performed with the approval of the animal experiment ethics committee at the University of Tokyo (approval number: 21–6) and according to the University of Tokyo’s guidelines for the care and use of laboratory animals. In human studies, the experimental protocol was approved by the Human Research Ethics Committee of the University of Tokyo (approval number: 24–3) and the Center for Information and Neural Networks (approval number: 1312260010). All participants were provided oral and written informed consents, and they signed the consent forms prior to each experiment.

### Animal Preparation for Recordings

Postnatal days (P) 28–35 male C57BL/6J mice (Japan SLC, Shizuoka, Japan) were used in the animal experiments as previously described in detail [78,79]. The animals were housed in cages in standard laboratory conditions (a 12-h light/dark cycle, free access to food and water). All efforts were made to minimize the animals' suffering and the number of animals used. The animals were anesthetized with ketamine (50 mg/kg, i.p.) and xylazine (10 mg/kg, i.p.). Anesthesia was confirmed by the lack of paw withdrawal, whisker movement, and eye blink reflexes. The head skin was then removed, and the animal was implanted with a metal head-holding plate. After 2 d of recovery, the head-fixation training on a custom-made stereotaxic fixture was repeated for 1−3 h per d until the implanted animal learned to remain quiet. During and after each session, the animal was rewarded with free access to sucrose-containing water. During the final three sessions, sham experiments were conducted to habituate the animal to the experimental conditions and noise. On the final 2−3 d, the animal was maintained virtually immobile, i.e., quiet but awake, for more than 2 h. After full habituation, the animals were anesthetized with ketamine/xylazine. A craniotomy (1 × 1 mm^2^), centered at 3.5 mm posterior to the bregma and 2.0 mm ventrolateral to the sagittal suture, was performed, and the dura was surgically removed. The exposed cortical surface was covered with 1.7–2.0% agar at a thickness of 0.5 mm. Throughout the experiments, a heating pad maintained the rectal temperature at 37°C, and 0.2% lidocaine was applied to the surgical region for analgesia. For patch-clamp recordings, the recorded area was confirmed by posthoc imaging of the intracellularly loaded Alexa 594, which was dissolved at 50 μM in patch-clamp solution. For calcium imaging, pressure-injected SR101, which was dissolved at 0.1 mM in Fura 2-containing solution, was imaged posthoc to confirm the recorded area. Recordings were initiated after recovery from anesthesia, which was confirmed by spontaneous whisker movements and touch-induced eye blink reflexes. The total periods of recording were restricted to less than 1 h to minimize stress in the animals.

### Visual Stimulation

Visual stimuli were generated in custom-written MATLAB routines (The MathWorks, Natick, MA, USA) with Psychtoolbox extensions. A 17-in TN-LCD monitor (refresh rate = 60 Hz) was placed 30 cm away from the right cornea, so that it covered 38.8° horizontally and 29.6° vertically of the mouse visual field. For flash stimulation, sinusoidal gratings (spatial frequency: 0.16 cpd; temporal frequency: 2 Hz; contrast: 100%) were presented in four evenly spaced orientations (0°, 45°, 90°, and 135°). The flash duration was set to range between 17–50 ms. Measurement using a high-speed CMOS camera (ORCA-Flash2.8, Hamamatsu, imaged at 2,000 Hz) revealed that a flashing light on the TN-LCD monitor decayed with a time constant τ_1/2_ = 5.5 ms, and thus, the afterglow was virtually ignorable. For each orientation, the gratings were presented at 2–4 spatial phases, and the responses were averaged to remove the effects of spatial phases. Flash stimuli were intervened with a gray screen for intervals of 8–10 s. In each set, stimuli with four orientations were presented in a pseudorandom order, and the set was repeated 10–40 times. For drifting grating stimulation, sinusoidal gratings (spatial frequency: 0.12 cpd; temporal frequency: 2 Hz; contrast: 100%) moved toward eight evenly spaced directions (0°, 45°, 90°, 135°, 180°, 225°, 270°, and 315°) for 1.5 s at intervals of 8–10 s for electrophysiology and for 2 s at an interval of 6 s for calcium imaging. A gray screen was shown during the interval period. In each set, drifting stimuli with eight directions were presented in a pseudorandom order, and the set was repeated 10–40 times. In the Flash+Drift trials, each flash stimulus was followed by a drifting grating stimulus at an SOA of 0.5 s. In S6 Fig, the flash stimuli were fixed at the vertical orientation (0°, vFlash), whereas in Fig 4 the drifting gratings were fixed at the vertical orientation (0°, 180°, vDrift) and moved rightward or leftward.

### VSD Imaging

The procedures for in vivo VSD imaging have been previously described in detail [33,80]. The dye RH-1692 (Optical Imaging, New York, NY) [81] was dissolved in 4-(2-hydroxyethyl)-1-piperazineethanesulphonic acid (HEPES)-buffered saline solution (0.6 mg ml^−1^) and applied to the exposed cortex for 60–90 min, which stained all neocortical layers. Imaging was initiated approximately 30 min after washing the unbound dye. To minimize movement artifacts because of respiration, the brain was covered with 1.5% agarose made in HEPES-buffered saline and sealed with a glass coverslip. For data collection, 12-bit images were captured at 6.67-ms temporal resolution with a charge-coupled device camera (1M60 Pantera, Dalsa, Waterloo, ON) and an EPIX E4DB frame grabber with XCAP 3.1 imaging software (EPIX, Inc., Buffalo Grove, IL). RH-1692 was excited with red LEDs (Luxeon K2, 627-nm center) and excitation filters of 630 ± 15 nm. Images were obtained with a microscope composed of front-to-front video lenses (8.6 × 8.6 mm field of view, 67 μm per pixel). The depth of field of our imaging setup was 1 mm. RH-1692 fluorescence was filtered through a 673-to-703-nm band-pass optical filter (Semrock, New York, NY). Visual responses were averaged from 40–80 trials of stimulus presentations. Responses to flashes were expressed as the percent change in RH-1692 fluorescence relative to the baseline fluorescence intensity (Δ*F*/*F*_0_ × 100%). Gating flashes were applied to the retina at a distance of approximately 10 cm from the cornea contralateral to the recording site to cover the entire optic angle. Stimulation was repeated every 10 s.

### Electrophysiology

The signal was amplified with a MultiClamp 700B, analyzed with pCLAMP10.1 (Molecular Devices, Union City, CA, USA) and digitized at 20 kHz. The data were reduced to 2 kHz and off-line analyzed using custom-written MATLAB routines. Patch-clamp recordings were obtained from L2/3 neurons at depths of 150–350 μm from the V1 surface using borosilicate glass electrodes (3.5–6.5 MΩ) that were pulled with a P-97 puller (Sutter Instruments, Novato, CA, USA). The electrode tips were lowered perpendicularly into the V1 with a DMX-11 electric manipulator (Narishige, Tokyo, Japan) or obliquely (at 30°) with a PatchStar micromanipulator (Scientifica, Uckfield, UK). For cell-attached recordings, pipettes were filled with aCSF. For whole-cell recordings, the intrapipette solution consisted of the following (in mM): 130 K-gluconate, 10 KCl, 10 HEPES, 10 Na_2_-phosphocreatine, 4 Mg-ATP, 0.3 Na_2_GTP, 0.05 Alexa-594 hydrazide, and 0.2% biocytin, adjusted to pH 7.3. For morphological reconstruction of the recorded cells, mice were perfused transcardially with 4% paraformaldehyde, and their brains were coronally sectioned at a thickness of 200 μm using a DTK-1500 vibratome (Dosaka, Kyoto, Japan). The sections were incubated with 0.3% H_2_O_2_ for 30 min and permeabilized with 0.2% Triton X-100 for 1 h. Then, the sections were processed with ABC reagent at 4°C overnight and developed with 0.0003% H_2_O_2_, 0.02% diaminobenzidine, and 10 mM (NH_4_)_2_Ni(SO_4_)_2_. Experiments in which the series resistance exceeded 70 MΩ or changed by more than 15% during the recording session were discarded. For each neuron, spike responses to a brief inward current were examined, and regular spiking neurons were selected as putative pyramidal cells for the subsequent analyses. LFPs were recorded at a depth of 300 μm from the V1 surface, which corresponded to L2/3, using borosilicate glass pipettes (1−2 MΩ) filled with aCSF. Traces were band-pass filtered between 1 and 250 Hz.

### Human EEG

Ten healthy adults (four males and six females, 25.9 ± 5.4 (mean ± SD) years old) with normal or corrected-to-normal vision participated in our EEG experiments. The EEG experiment was conducted in a dark room to explore early and late components of the visually evoked ERPs for brief exposures to high-contrast grating stimulus flashes. Visual stimuli were generated on a computer using Psychophysics MATLAB toolbox [82]. The stimuli were presented using a gamma-corrected [83] LCD display (EIZO FlexScan S2243W, EIZO corporation, Ishikawa, Japan) whose spatial resolutions were 1,920×1,200 pixels, and the refresh rate was 60 Hz. Participants viewed the stimuli at a 55-cm distance from the display. The experiment contained two stimulus conditions (vertical and horizontal gratings), and the EEG signals for each of the stimuli were acquired 200 times (100 for the horizontal grating and 100 for the vertical grating). In each trial, the start of the trial was informed by the change of the color of the central fixation point (from gray to white). After 3–4 s (randomly jittered to exclude participant’s expectation effect on the EEG signals) of the fixation color change, a high-contrast (100% from the gray background) grayscale sinusoidal grating (1.03 cycles per degree) pattern (35.2 × 24.4° in visual angle) was flashed for 50 ms. The background brightness was 17.80 cd/m^2^, which corresponds roughly to 4.88 lux, and the grating brightness ranged from 0.26 cd/m^2^ (0.07 lux) to 35.62 cd/m^2^ (9.77 lux). Then, participants were asked to keep fixating the central fixation for 4 s without blinking as much as possible. After the 4-s fixation period, the central fixation color changed from white to gray to inform the end of a trial. The task start was initiated by a button press by a participant. The participants could take breaks between trials as they liked, and they could proceed the experiments at their own paces. The stimulus presentation order was pseudorandomized for each participant. One EEG session took about 2 h. The human visual ERPs at O1 and O2 (following the international 10/20 coordinate convention) for the two stimulus configurations were collected at 1 kHz (the left earlobe was used as a reference) with a wireless EEG system (Polymate Mini AP108, Miyuki Giken Co., Ltd, Tokyo, Japan) with pasteless dry electrodes (National Institute of Information and Communications Technology, Japan) [84]. Electrode impedances for O1 and O2 were kept below 5 kΩ at the beginning of the measurements. Eye movements and blinks were simultaneously recorded with an electrode put on a left eye lid. The onset of the visual stimulus presentation and the EEG measurements were synchronized using a customized photo-trigger detection system (C6386, Hamamatsu Photonics K.K., Shizuoka, Japan). The recorded EEG and eye blink–related signals were saved on a computer using in-house MATLAB subroutines after each trial through a Bluetooth wireless connection. The ERP time series were analyzed using EEGLAB MATLAB toolbox ([85], http://sccn.ucsd.edu/eeglab/) and in-house subroutines written in MATLAB. The EEG signals were aligned offline so that we could evaluate the time series from −200 ms to 3,000 ms relative to the grating stimulus onset. The raw data were preprocessed offline by a linear trend removal and a band-pass filtering (0.5 to 100 Hz). Additionally, EEG epochs that contained large potentials exceeding the threshold (40 μV) and abnormal spike or drifting components were excluded by EEGLAB’s automatic outlier detection utilities and visual inspections. These noisy epochs were generally derived from eye movements and blinks. The signal amplitudes were recomputed carefully by taking the mean of −200 to 0 ms (relative to the stimulus onset) samples as the baseline for each epoch. The recorded signals from two electrodes were similar and hence averaged for each participant. Finally, the ERPs averaged over 10 participants were given as the final visual event-related time series. The statistical tests to explore whether the signals were higher or lower than the baseline were evaluated by the standard two-tailed *t* test at each sampling point (*p* < 0.05 without corrections of multiple comparisons).

### OSI and Tuning Curve

The OSI was defined according to the following equation:

$$\text{OSI}=\frac{\sqrt{{\left(\sum R_{\theta }\text{sin}2\theta \right)}^{2}+{\left(\sum R_{\theta }\text{cos}2\theta \right)}^{2}}}{\sum R_{\theta }}$$

where *R*_*θ*_ is the mean response amplitude to a grating with direction *θ* [86]. Note that this equation defines the normalized norm of the averaged vector [86] and may give a value that is different from OSI used in other reports [41]. The similarity of the tuning curves between the early and late responses was evaluated using the correlation coefficient (*R*) of the amplitudes of the responses:

$$R=\frac{\sum (R_{\theta \_\text{early}}-{\bar{R}}_{\theta \_\text{early}})\sum (R_{\theta \_\text{late}}-{\bar{R}}_{\theta \_\text{late}})}{\sqrt{\sum {\left(R_{\theta \_\text{early}}-{\bar{R}}_{\theta \_\text{early}}\right)}^{2}}\sqrt{\sum {\left(R_{\theta \_\text{late}}-{\bar{R}}_{\theta \_\text{late}}\right)}^{2}}}$$

where *R*_*θ*_early_ and *R*_*θ*_late_ are the amplitudes of early and late responses, respectively, to a grating with direction *θ*. ${\overset{-}{R}}_{\theta \_early}$ and ${\overset{-}{R}}_{\theta \_late}$ represent the mean of the response amplitudes *R*_*θ*_early_ and *R*_*θ*_late_ across all eight *θ*s. For each cell, the OSI and *R* were compared with their chance levels, which were estimated using a conventional random resampling method in which 1,000 surrogates were generated by randomly shuffling all trials irrespective of *θ*.

### Two-Photon Calcium Imaging

The mouse was placed in a stereotaxic frame and then on the stage of an upright microscope (BX61WI; Olympus). Cortical neurons were loaded with Fura 2, a calcium-sensitive fluorescent dye, under online visual guidance with a two-photon laser scanning microscope (FV1000; Olympus). Fura 2 AM was dissolved at 10 mM in DMSO with 10% pluronic acid and diluted at the final concentration of 1 mM in aCSF that contained 0.1 mM SR101. This solution was pressure-injected (50–100 mbar for 10 s) into V1 at a depth of 150–250 μm from the surface through a glass pipette (tip diameter: 10–30 μm). The pipette was carefully withdrawn, and the craniotomized area was sealed with 2% agar and a glass cover slip. After 50–70 min, which enabled the dye loading to the neuronal soma and the washout of extracellular dyes, the Fura-2 fluorescence was two-photon imaged from V1 L2/3 neurons. Neurons and astrocytes were discriminated based on astrocyte-specific staining with SR101 [87]. Fura 2 and SR101 were excited by a mode-locked Ti: sapphire laser at wavelengths of 800 nm and 910 nm, respectively (100 fs pulse width, 80 MHz pulse frequency; Maitai HP; Spectra Physics) [88]. Fluorescent light was corrected by a water-immersion objective lens (20×, numerical aperture 0.95; Olympus). Videos were taken from a 320×320-μm area at five frames per s using FV10-ASW software (version 3.0; Olympus). Neurons that exhibited significant visual responses above the baseline (*p* < 0.05, paired *t* test) in any recording session were selected for analysis.

### Virtual Optomotor System

The apparatus was located in a dark, soundproofed room. The room temperature was maintained at 25°C during the experiment. A virtual cylinder comprising a vertical sinusoidal grating (0.17 cpd, 10%–40% contrast) was displayed in three-dimensional coordinate space on four 24-in monitors (refresh rate: 60 Hz) that were arranged in a quadrangle arena. The images on the monitors were extended by two mirrors on the top and bottom of the arena. A platform (a white acrylic disc; *ϕ* = 6.0 cm) was positioned 13.5 cm above the bottom mirror. In each experiment, a single male P28–35 C57BL/6J mouse was placed on the platform and was allowed to move freely. The behavior of the mouse was monitored through a camera (Logicool HD Webcam C615; Logitech, Tokyo, Japan) that was attached over a small hole of the top mirror. Vertical gratings that drifted leftward or rightward (temporal frequency: 0.5 Hz) were presented simultaneously on all four screens for 2 s with a random interval between 2–4 s. From the animal’s point of view, the virtual cylinder appeared to rotate around the platform at an angular velocity of 5° per s). The mice normally tracked the grating with reflexive head movements in concert with the rotation direction. The drifting directions were randomly alternated, and the rotations were repeated 120 times in one session that took approximately 10 min. In some trials, either a vertical or horizontal grating (0.17 cycles per degree, 100% contrast) was flashed 0.5 or 3 s before a drifting grating. Animals were habituated to the system prior to the first behavioral test by experiencing at least one full session. When the mice slipped or jumped down from the platform during the test, they were manually returned to the platform, and the test was resumed. If the animal’s head tracked a cylinder rotation, the trial was counted as a “success.” Manual counting was checked by two independent trained researchers who were blind to the experimental conditions. Through computer-generated order randomization of the stimulation conditions, the experimenters were also blind to the treatment. The trials in which a mouse was grooming or made large movements were excluded from the analyses (invalid trials). The success rate was calculated as a ratio of the successful trials to the total valid trials. Tetrodotoxin was dissolved at 10 μM in aCSF and directly applied to the cortical surface 15 min prior to the behavioral sessions. The exposed cortices were covered with the craniotomized bone segments and mounted with dental cement. The effects of tetrodotoxin were confirmed by flash-induced LFP responses in V1 L2/3.

### Human Psychophysics

Eleven healthy right-handed individuals (three females) with normal or corrected-to-normal vision participated. The ages ranged from 22 to 42 years, with 26.5 ± 5.1 years (mean ± SD). The participants performed tasks using a computer mouse with their right hands. A 24-in monitor was placed at a distance of 0.5 m from the participants’ eyes in a dark, pseudosoundproofed room. The participants were instructed to report the motion direction of drifting gratings presented on the screen. A 2 × 2 cm^2^ open square was displayed at the center of the screen against a gray background (60 cd/m^2^, 5 lux). Each trial was initiated when a participant clicked the computer mouse on the square. Then, the square was filled in black, and after a random time interval between 1–3 s, a sinusoidal drifting grating (spatial frequency: 0.12 cpd; temporal frequency: 1 Hz; contrast: 40%) was presented for 0.25 s in one of four movement directions (0°, 90°, 180°, and 270°). A 50-ms beep tone was presented 0.5 s before a drifting grating stimulus. In some trials, a 50-ms grating flash (spatial frequency: 0.12 cpd; contrast: 100%) was displayed simultaneously with the tone. A full gray screen was displayed during all interstimulus intervals. After each stimulus, the participants were asked to move the mouse cursor in the same direction as the grating motion as rapidly as possible. When the mouse cursor traversed the edge of the square, the square became blank, which cued the trial completion. Incorrect motion reports or failures to respond within 600 ms (misses) from stimulus onset were considered errors and were indicated to the participants through a 200-ms peep tone. Each participant performed 160–244 trials per session.

## Supporting Information

S1 Data Flash-evoked biphasic responses in mouse V1 neurons. Excel spreadsheet containing the underlying numerical data for Fig 1. Raw response traces of all stimulus trials used in Fig 1A and 1B as well as mean response traces of individual neurons used in Fig 1D are contained.
(XLSX)

S2 Data Biphasic responses of field potentials in mouse and human visual cortex to grating flashes. Excel spreadsheet containing the underlying numerical data for Fig 2. Raw LFP and EEG recording traces of individual stimulus trials used in Fig 2A–2C are contained.
(XLSX)

S3 Data Orientation selectivity of late V1 response. Excel spreadsheet containing the underlying numerical data for Fig 3. Numerical values used to plot Fig 3A–3F and raw membrane potential traces of individual stimulus trials used in Fig 3C are contained.
(XLSX)

S4 Data Flash-induced facilitation of *V*_*m*_ response to subsequent visual information. Excel spreadsheet containing the underlying numerical data for Fig 4. Membrane potential traces used to plot Fig 4B and individual values used to plot means ± SEMs of Fig 4C–4E are contained.
(XLSX)

S5 Data Flash-enhanced visual perception in mice and humans. Excel spreadsheet containing the underlying numerical data for Fig 5. Individual values used to plot means ± SEMs of Fig 5B, 5C, 5E, and 5F are contained.
(XLSX)

S6 Data Characterization of biphasic *V*_m_ responses to flashes. Excel spreadsheet containing the underlying numerical data for S1 Fig. Raw membrane potential traces of individual stimulus trials used in S1B Fig and numerical values used to plot S1C Fig are contained.
(XLSX)

S7 Data Spatiotemporal patterns of flash-evoked neocortical activity. Excel spreadsheet containing the underlying numerical data for S2 Fig. Raw VSD signals of all animals used in S2A Fig, raw LFP traces of individual stimulus trials used in S2B Fig, and numerical values used to plot S2C Fig are contained.
(XLSX)

S8 Data Contrast dependence of flash-induced subthreshold *V*_m_ responses. Excel spreadsheet containing the underlying numerical data for S3 Fig. Membrane potential traces used to plot S3A Fig and individual values used to plot means ± SEMs of S3B Fig are contained.
(XLSX)

S9 Data Orientation selectivity of flash-induced visual responses. Excel spreadsheet containing the underlying numerical data for S4 Fig. Individual values used to plot S4A–S4C Fig are contained.
(XLSX)

S10 Data Two-photon calcium imaging of flash-induced V1 responses. Excel spreadsheet containing the underlying numerical data for S5 Fig. Raw and mean recording traces used in S5C and S5E Fig, individual values used to plot means ± SEMs of S5B and S5E Fig and individual values used to plot S5F and S5G Fig are contained.
(XLSX)

S11 Data Flash-enhanced V1 response to an identical orientation: multineuron calcium imaging. Excel spreadsheet containing the underlying numerical data for S6 Fig. Raw and mean recording traces used in S6B Fig, individual values used to plot means ± SEMs of S6B and S6C Fig, and individual values used to plot S6D and S6E Fig are contained.
(XLSX)

S12 Data Effect of local application of tetrodotoxin to V1 on mouse head-tracking responses. Excel spreadsheet containing the underlying numerical data for S7 Fig. Raw LFP traces of individual stimulus trials used in S7A Fig and individual values used to plot means ± SEMs of S7B Fig are contained.
(XLSX)

S13 Data Effect of phase differences between two flashes on motion perception. Excel spreadsheet containing the underlying numerical data for S8 Fig. Individual values used to plot means ± SEMs of S8B Fig are contained.
(XLSX)

S1 Fig Characterization of biphasic *V*_m_ responses to flashes. (A) The left photo shows a coronal slice indicating the recorded site marked by pressure application of Alexa 594 loaded in the patch pipette, which corresponds to the V1 monocular region. The right photo shows posthoc reconstruction of a whole-cell–recorded L2 pyramidal neuron with intracellular biocytin staining. (B) *V*_*m*_ responses to 0.05-s full-field grating flashes were whole-cell recorded from V1 L2/3 neurons of awake mice. Raw traces at 10 consecutive trials (*top*) and the mean ± SD (*bottom*) of the subthreshold *V*_*m*_ responses of cell #12 in 40 trials. The voltage response consisted of an early depolarization (E) that occurred earlier than 0.3 s after the stimulus onset and a late depolarization (L) that persisted up to 2 s and had a peak at approximately 0.4–2.0 s. (C) The area under the curve of late depolarization was plotted against the peak amplitudes of the early (left) and late (middle) depolarizations and the peak latency of late depolarizations (right). Each purple circle indicates a single cell, and the gray symbols indicate the means ± SDs of 28 neurons. The dashed line represents the best linear fit. All neurons exhibited significant early and late depolarizations (*p* < 0.05, paired *t* test, calculated by the peak and mean amplitude, respectively, for the early and late response periods). (D) Pie charts indicate the distributions of cells classified as early responsive (E), late responsive (L), or early and late responsive (E + L), or the other nonresponsive cells for spike (top) and subthreshold *V*_*m*_ responses (bottom). All recorded neurons (*n* = 28 cells) showed significant early and late subthreshold *V*_*m*_ responses (E + L), but their firing response types varied.
(TIF)

S2 Fig Spatiotemporal patterns of flash-evoked neocortical activity. VSD signal was time-lapse imaged from the right hemisphere, while a full-field grating flash was presented to the contralateral eye. (A) The top-left schematic indicates the cortical regions, including the V1. The snapshots indicate a time series of representative images at times indicated below. The bottom traces demonstrate the line-scanned VSD signal along the anterior–posterior axis of the cortex relative to V1, indicated in the red line in the top-left VSD image. Scale bar = 2 mm. (B) Mean ± SEM of VSD signals in V1 and retrosplenial cortex (RS). *n* = 8 mice. (C) V1 VSD signals were fitted to dual Gaussian curves. Left: Two representative fittings. Right: In all eight mice tested, *R* ^2^ exhibited *p* < 0.01.
(TIF)

S3 Fig Contrast dependence of flash-induced subthreshold *V*_m_ responses. (A) Representative *V*_m_ traces after flash stimulation of gratings with contrasts of 100%, 50%, 25%, or 10%. Whole-cell recordings were acquired from V1 L2/3 neurons in awake mice, whose contralateral eyes were presented with 0.05-s grating flashes. (B) Mean ± SEM of the amplitudes of early and late *V*_*m*_ responses as a function of grating contrast. (*n* = 7 cells from 7 mice).
(TIF)

S4 Fig Orientation selectivity of flash-induced visual responses. Responses to 0.05-s full-field grating flashes were recorded from V1 L2/3 neurons by patch-clamp technique. (A) The relationship between the firing rate and OSI for individual visual responsive neurons (*p* = 0.490, *R* ^2^ = 0.01, *n* = 44 cells). (B) The representative spike and *V*_*m*_ responses a V1 L2/3 neuron (Cell 1) and its orientation tuning curves of the firing rate (black) and subthreshold *V*_*m*_ responses (gray) are plotted. (C) Mean (black line) and each (gray circle) OSI of the subthreshold *V*_*m*_ responses and firing rates. The OSIs of the spike responses were significantly higher compared with the subthreshold *V*_*m*_ responses (*p* = 0.005, *t*_19_ = 3.17, *n* = 20 cells, paired *t* test).
(TIF)

S5 Fig Two-photon calcium imaging of flash-induced V1 responses. (A) Calcium activity from mouse V1 L2/3 neurons was imaged using a two-photon laser microscope. Fura 2 AM, a fluorescence calcium indicator, was locally applied to V1 L2/3. The photograph indicates a two-photon image of a Fura 2-labelled V1 L2/3 neuron. Simultaneous recordings of spikes by cell-attached and calcium imaging techniques were performed on the neuron. The shadow of the patch pipette is outlined by two white dashed lines. (B) The amplitude of the calcium signal (*|*Δ*F*/*F|*) was plotted against the number of cell-attached recorded spikes with a time window of 500 ms. Data represent the means ± SEMs of 5 cells. (C) Individual spikes (top trace recorded in the cell-attached patch-clamp configuration) with the minimal interspike interval of 372 ms could be distinguished by different onsets of calcium transients recorded from the soma. Note that a calcium rise decreases the two-photon fluorescence of Fura 2. (D) The photograph indicates a typical two-photon image of Fura 2-labelled V1 L2/3 neurons. (E) The left traces indicate raw (gray) and mean (black) Δ*F*/*F* of an example cell marked by the arrowhead in (D). The timing and pattern of visual stimuli are indicated above the traces. The right plot indicates the orientation tuning curve of |Δ*F*/*F*| in the same neuron. Error bars represent the SEMs of 12 trials. The baseline is indicated by a pink dotted line. For each stimulus orientation, statistical analyses (*p < 0.05 versus baseline, *n* = 10–18 trials, paired *t* test) were conducted to determine whether the |Δ*F*/*F*| amplitude was significantly higher than the baseline |Δ*F*/*F*| fluctuations. (F) The top pie chart shows the distribution of cells classified into cells that showed significant |Δ*F*/*F*| responses for at least one orientation (responsive) and cells that showed no activity changes. The bottom bar graph shows the distribution of responsive cells across preferred orientations. (G) The cumulative probability distribution of the |Δ*F*/*F*| OSIs of the 323 responsive cells compared with the late-spiking OSIs of the 31 cells in patch-clamp recordings (*p* = 0.497, Kolmogorov-Smirnov test).
(TIF)

S6 Fig Flash-enhanced V1 response to an identical orientation: multineuron calcium imaging. (A) A schematic shows the visual stimulation protocol without (Drift-only, control) and with (Flash+Drift) 0.05-s full-field grating flashes followed by 2-s drifting-grating stimulus (Drift) presented 0.5 s after vertically grating flashes (0°, vFlash). Drift-only and Flash+Drift trials were applied in a random order, and the responses were compared to measure how the preceding vFlash modulated the Δ*F*/*F* response to Drift with eight directions. (B) Neuronal responses to visual stimuli were recorded using two-photon calcium imaging. The left panel indicates raw Δ*F*/*F* traces at Drift-only trials (gray) and Flash+Drift trials (black) in cell #155. The timing and pattern of visual stimulation are indicated above the traces. The stimulus combination was described as Δorientation, which indicates the orientation difference between the Drift and vFlash. The right plot is the orientation tuning curve of the mean |Δ*F*/*F*| in the same neuron. Error bars represent the SEMs of 14 trials. Drift-only and Flash+Drift trials are shown in gray and black, respectively. The baseline is indicated by the pink dashed line. For each stimulus orientation, statistical analyses (*p < 0.05 versus baseline, *n* = 10–18 trials, paired *t* test) were conducted to determine whether the |Δ*F*/*F*| amplitude was significantly higher compared with the baseline Δ*F*/*F* fluctuation. Dark red boxes below the tuning plot indicate significant responses, whereas open boxes indicate nonsignificant responses. (C) Three other examples of the |Δ*F*/*F*| orientation tuning curves and the statistical results. (D) Data are summarized from 581 cells. For each Δorientation, the numbers of cells that exhibited significant |Δ*F*/*F*| responses between Drift-only and Flash+Drift trials were compared (*n* = 581 cells from 11 mice). More cells became responsive at Δorientation = 0°. (E) The data analyzed in (D) was resolved based on the orientation preferences of individual neurons.
(TIF)

S7 Fig Effect of local application of tetrodotoxin to V1 on mouse head-tracking responses. (A) Mean ± SD of LFPs were recorded from V1 before (top) and 10 min after (bottom) local application of tetrodotoxin (TTX, 10 μM) to the V1 surface. Tetrodotoxin blocked flash-induced LFP responses. This effect lasted more than 120 min after the TTX application. (B) The tracking rates in vDrift-only (-) and Δorientation = 0° trials were measured 50–95 min after the TTX application (*n* = 4 mice).
(TIF)

S8 Fig Effect of phase differences between two flashes on motion perception. (A) Schematic of the behavioral procedure of a visual motion detection task in humans. In each trial, either vertical or horizontal flash was presented 0.5 s before in one video frame of another flash (0.017 s) to which the participants were required to respond "the motion direction" by pressing a left or right arrow key. The phase of the grating for two flashes was randomized to examine whether the phase shift would induce a motion perception. (B) The correct response rate of the participants did not differ from the chance level, i.e., 50% (*p* = 0.254, *F*_4,40_ = 1.39, *n* = 5 participants, two-way ANOVA), which indicates that the phase shift between two flashes did not induce motion illusion.
(TIF)

## Abbreviations

AM: acetoxymethyl ester
EEG: electroencephalogram
ERP: event-related potential
LFP: local field potential
LGN: lateral geniculate nucleus
OSI: orientation selectivity index
SD: standard deviation
SEM: standard error of the mean
SOA: stimulus-onset asynchrony
*V*_*m*_: membrane voltage
VSD: voltage-sensitive dye
V1: primary visual cortex
