# Supplementary figures and images for: Neocortical Rebound Depolarization Enhances Visual Perception
Source: PLoS Biol. 2015 Aug 14;13(8):e1002231. doi: 10.1371/journal.pbio.1002231 (PMC4537103; doi:10.1371/journal.pbio.1002231)

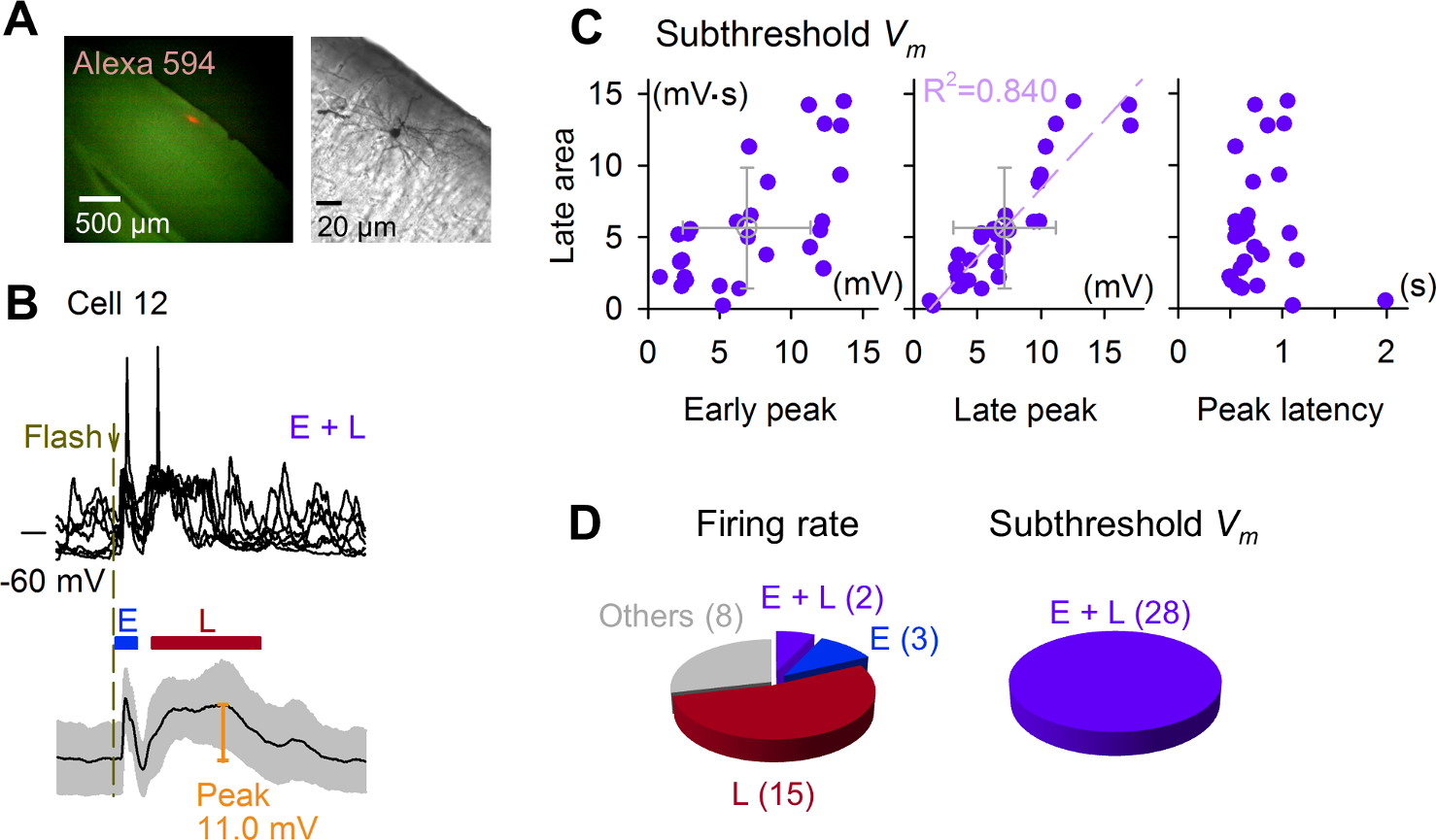

Supplement: S1 Fig — (A) The left photo shows a coronal slice indicating the recorded site marked by pressure application of Alexa 594 loaded in the patch pipette, which corresponds to the V1 monocular region. The right photo shows posthoc reconstruction of a whole-cell–recorded L2 pyramidal neuron with intracellular biocytin staining. (B) V m responses to 0.05-s full-field grating flashes were whole-cell recorded from V1 L2/3 neurons of awake mice. Raw traces at 10 consecutive trials (top) and the mean ± SD (bottom) of the subthreshold V m responses of cell #12 in 40 trials. The voltage response consisted of an early depolarization (E) that occurred earlier than 0.3 s after the stimulus onset and a late depolarization (L) that persisted up to 2 s and had a peak at approximately 0.4–2.0 s. (C) The area under the curve of late depolarization was plotted against the peak amplitudes of the early (left) and late (middle) depolarizations and the peak latency of late depolarizations (right). Each purple circle indicates a single cell, and the gray symbols indicate the means ± SDs of 28 neurons. The dashed line represents the best linear fit. All neurons exhibited significant early and late depolarizations (p < 0.05, paired t test, calculated by the peak and mean amplitude, respectively, for the early and late response periods). (D) Pie charts indicate the distributions of cells classified as early responsive (E), late responsive (L), or early and late responsive (E + L), or the other nonresponsive cells for spike (top) and subthreshold V m responses (bottom). All recorded neurons (n = 28 cells) showed significant early and late subthreshold V m responses (E + L), but their firing response types varied. (TIF) [file pbio.1002231.s014.TIF]

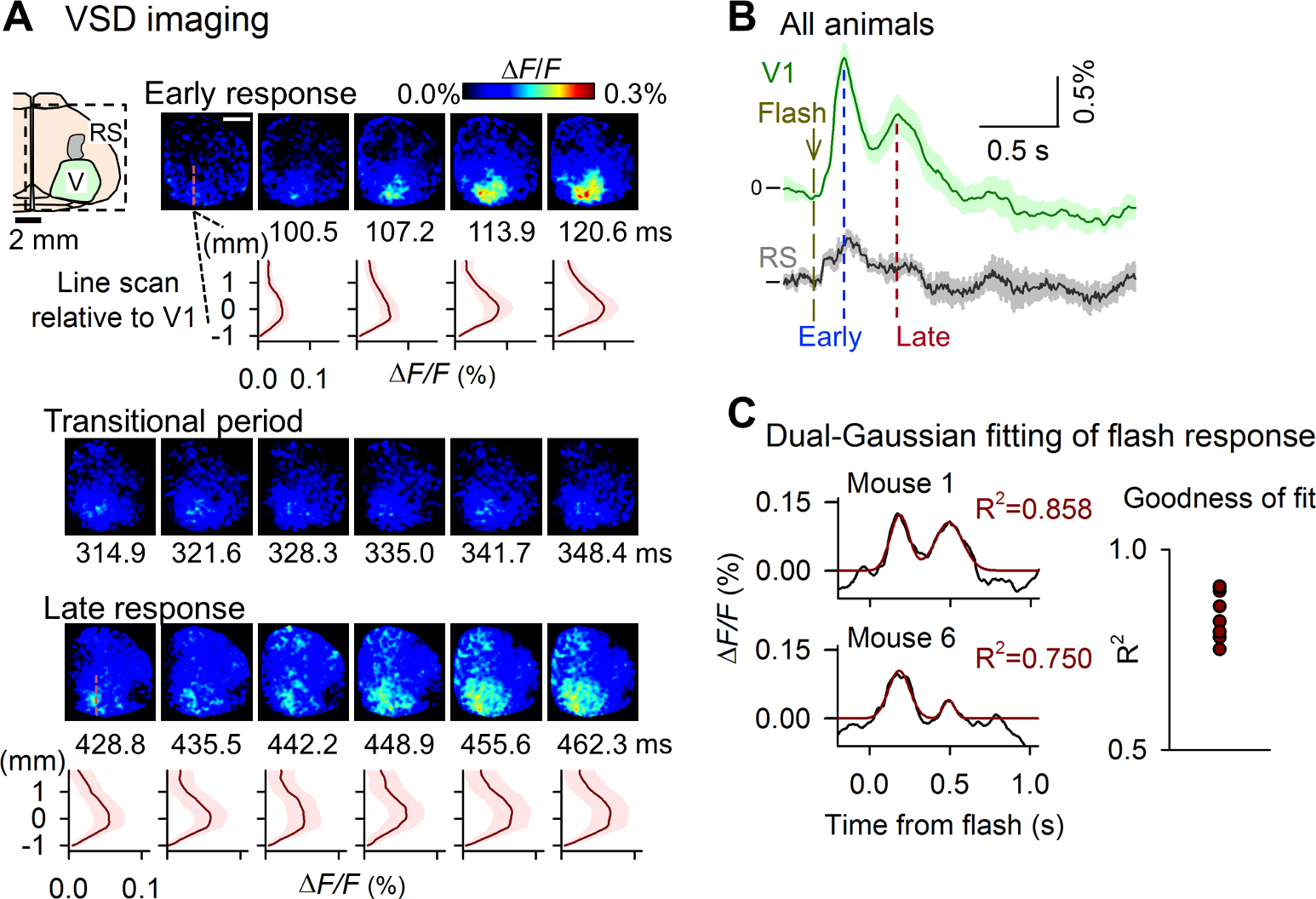

Supplement: S2 Fig — VSD signal was time-lapse imaged from the right hemisphere, while a full-field grating flash was presented to the contralateral eye. (A) The top-left schematic indicates the cortical regions, including the V1. The snapshots indicate a time series of representative images at times indicated below. The bottom traces demonstrate the line-scanned VSD signal along the anterior–posterior axis of the cortex relative to V1, indicated in the red line in the top-left VSD image. Scale bar = 2 mm. (B) Mean ± SEM of VSD signals in V1 and retrosplenial cortex (RS). n = 8 mice. (C) V1 VSD signals were fitted to dual Gaussian curves. Left: Two representative fittings. Right: In all eight mice tested, R 2 exhibited p < 0.01. (TIF) [file pbio.1002231.s015.TIF]

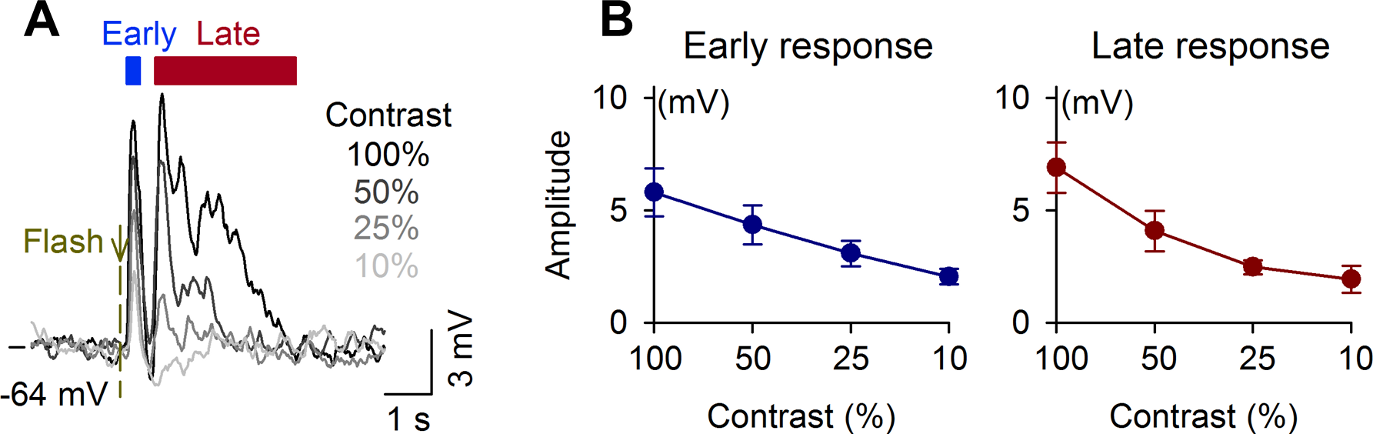

Supplement: S3 Fig — (A) Representative V m traces after flash stimulation of gratings with contrasts of 100%, 50%, 25%, or 10%. Whole-cell recordings were acquired from V1 L2/3 neurons in awake mice, whose contralateral eyes were presented with 0.05-s grating flashes. (B) Mean ± SEM of the amplitudes of early and late V m responses as a function of grating contrast. (n = 7 cells from 7 mice). (TIF) [file pbio.1002231.s016.TIF]

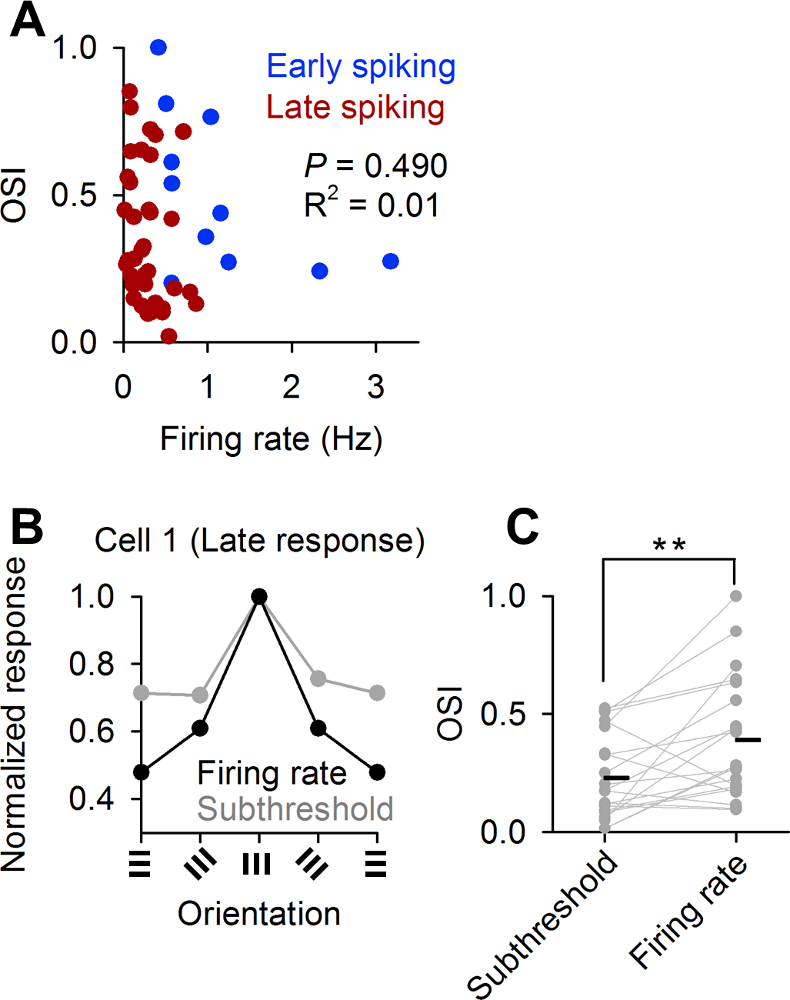

Supplement: S4 Fig — Responses to 0.05-s full-field grating flashes were recorded from V1 L2/3 neurons by patch-clamp technique. (A) The relationship between the firing rate and OSI for individual visual responsive neurons (p = 0.490, R 2 = 0.01, n = 44 cells). (B) The representative spike and V m responses a V1 L2/3 neuron (Cell 1) and its orientation tuning curves of the firing rate (black) and subthreshold V m responses (gray) are plotted. (C) Mean (black line) and each (gray circle) OSI of the subthreshold V m responses and firing rates. The OSIs of the spike responses were significantly higher compared with the subthreshold V m responses (p = 0.005, t 19 = 3.17, n = 20 cells, paired t test). (TIF) [file pbio.1002231.s017.TIF]

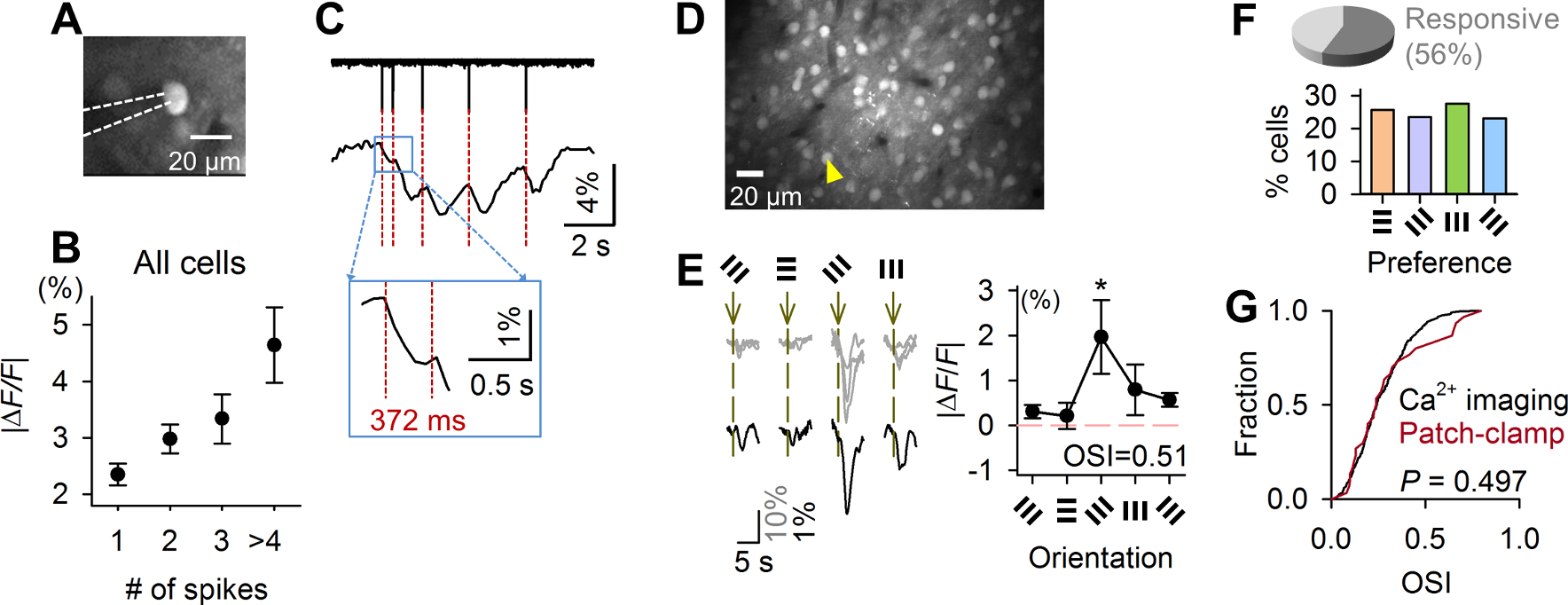

Supplement: S5 Fig — (A) Calcium activity from mouse V1 L2/3 neurons was imaged using a two-photon laser microscope. Fura 2 AM, a fluorescence calcium indicator, was locally applied to V1 L2/3. The photograph indicates a two-photon image of a Fura 2-labelled V1 L2/3 neuron. Simultaneous recordings of spikes by cell-attached and calcium imaging techniques were performed on the neuron. The shadow of the patch pipette is outlined by two white dashed lines. (B) The amplitude of the calcium signal (|ΔF/F|) was plotted against the number of cell-attached recorded spikes with a time window of 500 ms. Data represent the means ± SEMs of 5 cells. (C) Individual spikes (top trace recorded in the cell-attached patch-clamp configuration) with the minimal interspike interval of 372 ms could be distinguished by different onsets of calcium transients recorded from the soma. Note that a calcium rise decreases the two-photon fluorescence of Fura 2. (D) The photograph indicates a typical two-photon image of Fura 2-labelled V1 L2/3 neurons. (E) The left traces indicate raw (gray) and mean (black) ΔF/F of an example cell marked by the arrowhead in (D). The timing and pattern of visual stimuli are indicated above the traces. The right plot indicates the orientation tuning curve of |ΔF/F| in the same neuron. Error bars represent the SEMs of 12 trials. The baseline is indicated by a pink dotted line. For each stimulus orientation, statistical analyses (*p < 0.05 versus baseline, n = 10–18 trials, paired t test) were conducted to determine whether the |ΔF/F| amplitude was significantly higher than the baseline |ΔF/F| fluctuations. (F) The top pie chart shows the distribution of cells classified into cells that showed significant |ΔF/F| responses for at least one orientation (responsive) and cells that showed no activity changes. The bottom bar graph shows the distribution of responsive cells across preferred orientations. (G) The cumulative probability distribution of the |ΔF/F| OSIs of the 323 responsive cells [file pbio.1002231.s018.TIF]

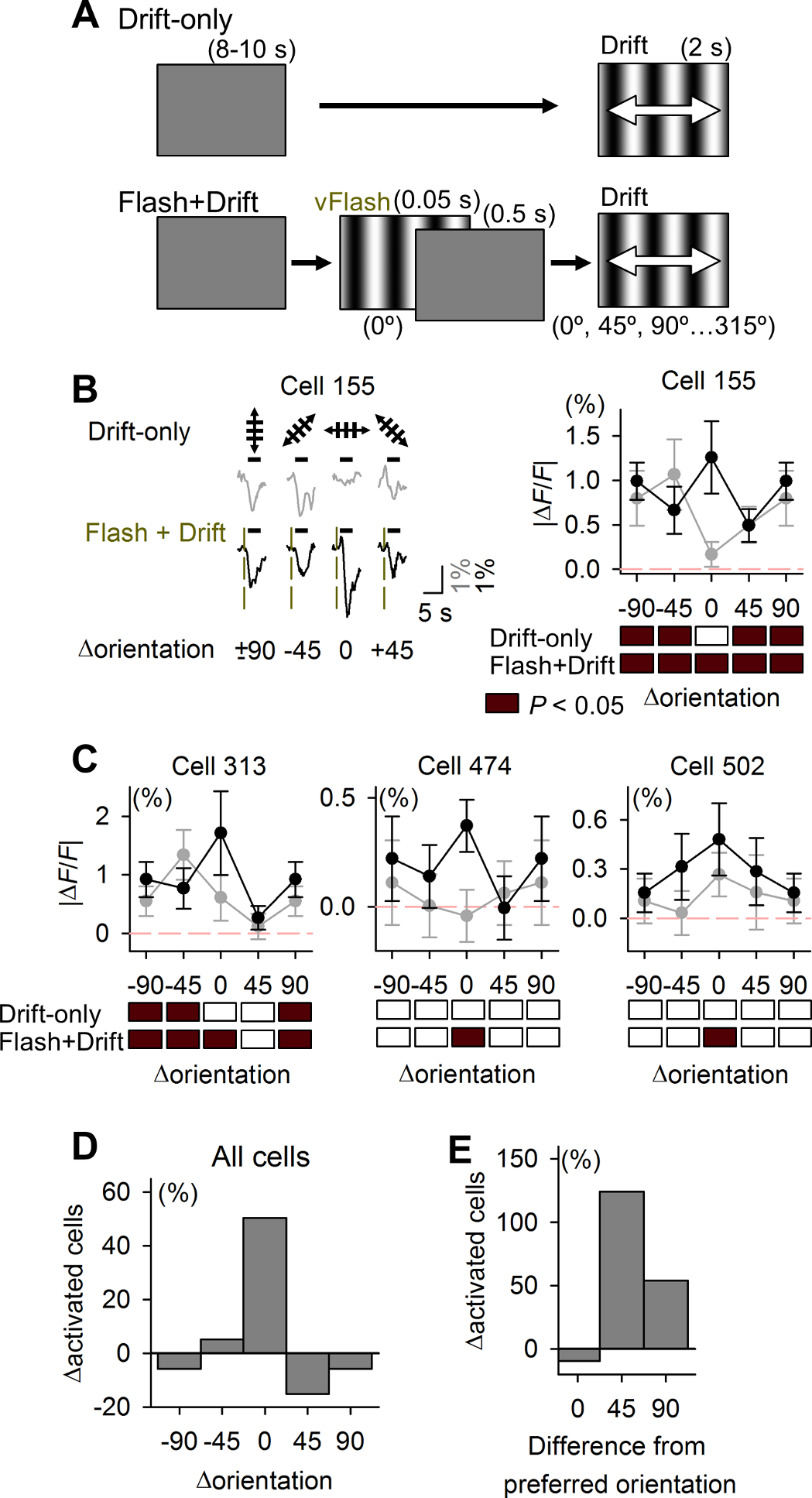

Supplement: S6 Fig — (A) A schematic shows the visual stimulation protocol without (Drift-only, control) and with (Flash+Drift) 0.05-s full-field grating flashes followed by 2-s drifting-grating stimulus (Drift) presented 0.5 s after vertically grating flashes (0°, vFlash). Drift-only and Flash+Drift trials were applied in a random order, and the responses were compared to measure how the preceding vFlash modulated the ΔF/F response to Drift with eight directions. (B) Neuronal responses to visual stimuli were recorded using two-photon calcium imaging. The left panel indicates raw ΔF/F traces at Drift-only trials (gray) and Flash+Drift trials (black) in cell #155. The timing and pattern of visual stimulation are indicated above the traces. The stimulus combination was described as Δorientation, which indicates the orientation difference between the Drift and vFlash. The right plot is the orientation tuning curve of the mean |ΔF/F| in the same neuron. Error bars represent the SEMs of 14 trials. Drift-only and Flash+Drift trials are shown in gray and black, respectively. The baseline is indicated by the pink dashed line. For each stimulus orientation, statistical analyses (*p < 0.05 versus baseline, n = 10–18 trials, paired t test) were conducted to determine whether the |ΔF/F| amplitude was significantly higher compared with the baseline ΔF/F fluctuation. Dark red boxes below the tuning plot indicate significant responses, whereas open boxes indicate nonsignificant responses. (C) Three other examples of the |ΔF/F| orientation tuning curves and the statistical results. (D) Data are summarized from 581 cells. For each Δorientation, the numbers of cells that exhibited significant |ΔF/F| responses between Drift-only and Flash+Drift trials were compared (n = 581 cells from 11 mice). More cells became responsive at Δorientation = 0°. (E) The data analyzed in (D) was resolved based on the orientation preferences of individual neurons. (TIF) [file pbio.1002231.s019.TIF]

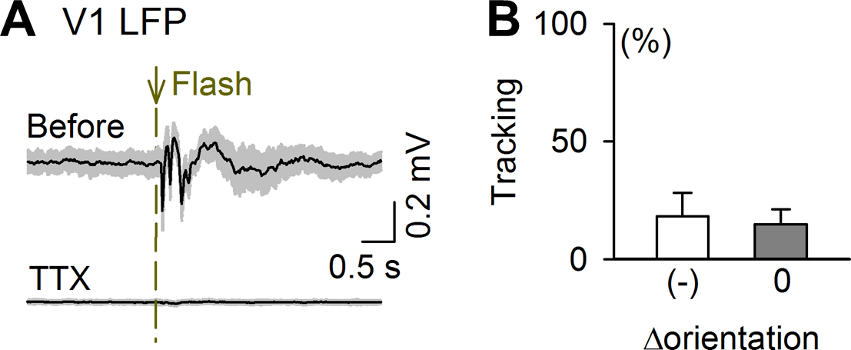

Supplement: S7 Fig — (A) Mean ± SD of LFPs were recorded from V1 before (top) and 10 min after (bottom) local application of tetrodotoxin (TTX, 10 μM) to the V1 surface. Tetrodotoxin blocked flash-induced LFP responses. This effect lasted more than 120 min after the TTX application. (B) The tracking rates in vDrift-only (-) and Δorientation = 0° trials were measured 50–95 min after the TTX application (n = 4 mice). (TIF) [file pbio.1002231.s020.TIF]

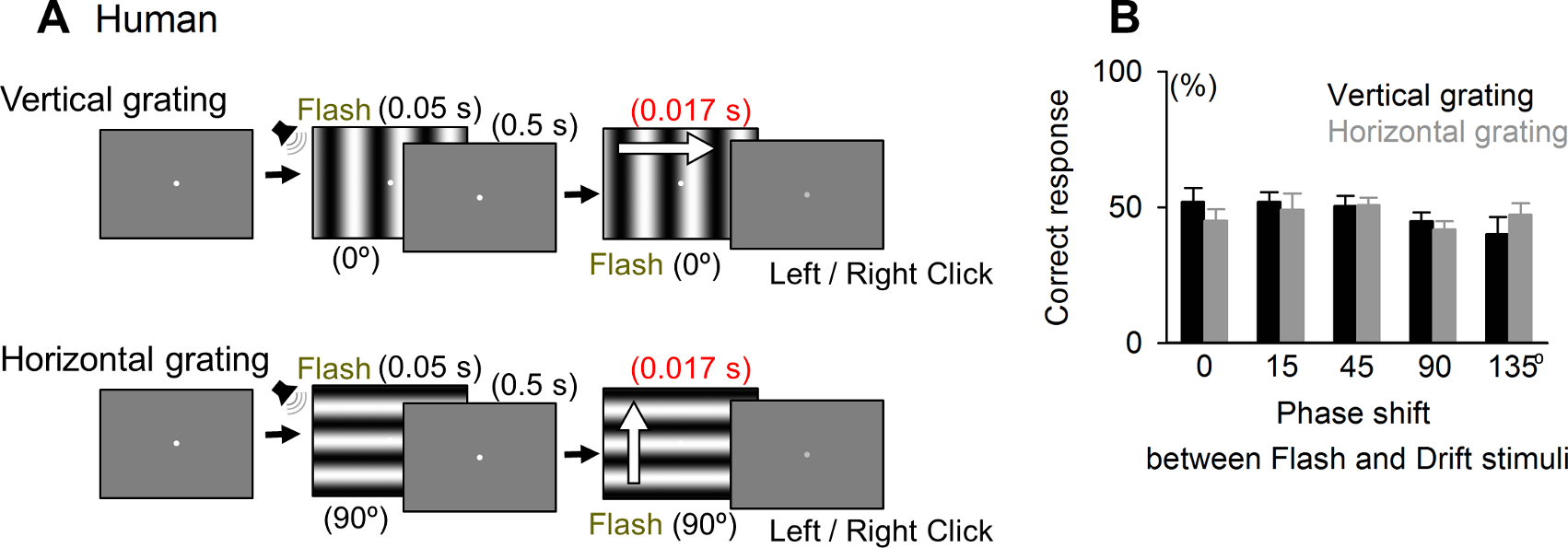

Supplement: S8 Fig — (A) Schematic of the behavioral procedure of a visual motion detection task in humans. In each trial, either vertical or horizontal flash was presented 0.5 s before in one video frame of another flash (0.017 s) to which the participants were required to respond "the motion direction" by pressing a left or right arrow key. The phase of the grating for two flashes was randomized to examine whether the phase shift would induce a motion perception. (B) The correct response rate of the participants did not differ from the chance level, i.e., 50% (p = 0.254, F 4,40 = 1.39, n = 5 participants, two-way ANOVA), which indicates that the phase shift between two flashes did not induce motion illusion. (TIF) [file pbio.1002231.s021.TIF]
